# Crystal structures of the EVE-HNH endonuclease VcaM4I in the presence and absence of DNA

**DOI:** 10.1093/nar/gkaa1218

**Published:** 2021-01-15

**Authors:** Michal Pastor, Honorata Czapinska, Igor Helbrecht, Katarzyna Krakowska, Thomas Lutz, Shuang-yong Xu, Matthias Bochtler

**Affiliations:** International Institute of Molecular and Cell Biology, Trojdena 4, 02-109 Warsaw, Poland; Institute of Biochemistry and Biophysics, Polish Academy of Sciences, Pawinskiego 5a, 02-106 Warsaw, Poland; International Institute of Molecular and Cell Biology, Trojdena 4, 02-109 Warsaw, Poland; International Institute of Molecular and Cell Biology, Trojdena 4, 02-109 Warsaw, Poland; Institute of Biochemistry and Biophysics, Polish Academy of Sciences, Pawinskiego 5a, 02-106 Warsaw, Poland; International Institute of Molecular and Cell Biology, Trojdena 4, 02-109 Warsaw, Poland; New England Biolabs, Inc. 240 County Road, Ipswich, MA 01938, USA; New England Biolabs, Inc. 240 County Road, Ipswich, MA 01938, USA; International Institute of Molecular and Cell Biology, Trojdena 4, 02-109 Warsaw, Poland; Institute of Biochemistry and Biophysics, Polish Academy of Sciences, Pawinskiego 5a, 02-106 Warsaw, Poland

## Abstract

Many modification-dependent restriction endonucleases (MDREs) are fusions of a PUA superfamily modification sensor domain and a nuclease catalytic domain. EVE domains belong to the PUA superfamily, and are present in MDREs in combination with HNH nuclease domains. Here, we present a biochemical characterization of the EVE-HNH endonuclease VcaM4I and crystal structures of the protein alone, with EVE domain bound to either 5mC modified dsDNA or to 5mC/5hmC containing ssDNA. The EVE domain is moderately specific for 5mC/5hmC containing DNA according to EMSA experiments. It flips the modified nucleotide, to accommodate it in a hydrophobic pocket of the enzyme, primarily formed by P24, W82 and Y130 residues. In the crystallized conformation, the EVE domain and linker helix between the two domains block DNA binding to the catalytic domain. Removal of the EVE domain and inter-domain linker, but not of the EVE domain alone converts VcaM4I into a non-specific toxic nuclease. The role of the key residues in the EVE and HNH domains of VcaM4I is confirmed by digestion and restriction assays with the enzyme variants that differ from the wild-type by changes to the base binding pocket or to the catalytic residues.

## Graphical Abstract

**Graphical Abstract:**
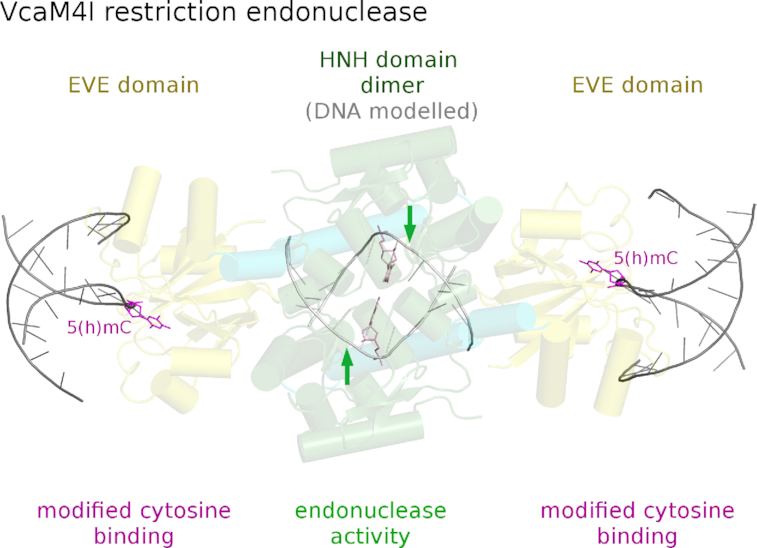
Crystal structures of VcaM4I restriction endonuclease provide the first structural description of the EVE domain-modified DNA interaction. Each protomer of the dimeric enzyme is composed of EVE domain responsible for modification detection and HNH domain responsible for DNA cleavage. The EVE domains flip the modified cytosine bases and bind them in dedicated hydrophobic pockets.

## INTRODUCTION

Most restriction modification systems use methylation as a mark of ‘self’ and target non-modified DNA. Such systems are typically sequence specific and include a methyltransferase (MTase) that protects DNA by methylation in the context relevant for cleavage. Perhaps in response to the wide-spread occurrence of ‘canonical’ restriction systems, many phages have modified bases in their genomes that protect against this defense. Modification dependent restriction endonucleases (MDREs) specifically target DNA that is modified, and are non-toxic to a host with non-modified DNA. Therefore, they are ‘orphan’ in the sense that they come without accompanying MTases, but unlike other restriction modification systems, they are often present in defense islands.

MDREs that do not depend on nucleoside triphosphates (NTPs) are typically encoded by single open reading frames, and are modular proteins that consist of separate modification-sensing and nuclease domains. Those studied to date have a preference for 5-methylcytosine (5mC), 5-hydroxymethylcytosine (5hmC), glucosyl-5-hydroxymethylcytosine (g5hmC) or 6-methyladenine (6mA). The well-characterized examples include endonucleases of the SRA-PD-(D/E)XK (MspJI) (1), PD-(D/E)XK-SRA (PvuRts1I) (2), SRA-HNH (TagI) (3), NEco-HNH (EcoKMcrA) (4) and PD-(D/E)XK-wH (DpnI, wH, winged helix domain) types (5). With the exception of the latter two families, the NTP-independent MDREs use an SRA or related domain for the detection of DNA modifications. The SRA domains characterized to date are specific for cytosine modifications (6,7). They extrude the modified cytosine residue from the stack of DNA bases, and accommodate it in a dedicated and highly conserved pocket (8–10). In both prokaryotes and eukaryotes, cytosine modifications often occur in palindromic or nearly palindromic contexts. In such cases, SRA domains are typically either insensitive to modifications in the other strand or discriminate against DNA that is modified in both strands (9). Unlike for MBD (11) or NEco domains (12), specificity for DNA methylation in both strands arises only as a consequence of cooperation of two SRA domains in a dimer context (13).

SRA domains, known for modified DNA binding, belong to the larger PUA superfamily of domains, named after pseudouridine synthase and archaeosine transglycosylase (14). The superfamily comprises several other domain families, including YTH and EVE domains, that are so far predominantly associated with binding of modified RNA nucleobases (15,16), despite hints that point to a role in DNA biology (17,18). Recently, it has become clear that in many eu- and archaebacteria, PUA superfamily domains cooperate with PD-(D/E)XK or HNH nuclease domains, either as fused proteins (19) or in dedicated protein complexes (18). The fusion proteins could be directed against RNA or DNA. So far, experimental evidence for RNA-directed activity is lacking, but there is considerable support for DNA-directed activity. A role of the enzymes as MDREs is consistent with the spotty phylogenetic distribution and a lack of accompanying DNA MTases. It is also supported by lack of toxicity to unprotected hosts, (moderate) modification dependent DNA endonuclease activity *in vitro*, and (moderate) modification dependent restriction activity *in vivo* (18,19).

Among the MDREs with modification sensing PUA superfamily domains, those with SRA (3), SRA-like (2), and YTH domains (18) have been characterized in more detail already. By contrast, little attention has so far been paid to the recently discovered MDREs with an EVE domain as the modification sensor. The group of EVE-HNH MDREs includes VcaM4I, TspA15I and CmeDI. TspA15I requires divalent cations as co-factors. *In vitro*, its activity is higher with Mn^2+^ than with Mg^2+^ ions. In high concentration the enzyme cleaves any DNA, but dilution series demonstrate a preference for 5hmC over g5hmC containing or unmodified DNA (19). Consistently, TspA15I expression in *E. coli* cells protected against T4gt phage (which due to glucosyltransferase deficiency contains 5hmC), with lower efficiency against T4 phage (which contains g5hmC), but not against λvir phage (19). Structures that could shed light on the mode of binding of the EVE domain to DNA containing modified cytosine bases are not available. Among several EVE-HNH MDREs, VcaM4I from *Vibrio campbellii* was the only one that yielded well-diffracting crystals. Therefore, we focused our attention on VcaM4I as the prototype for this group of enzymes.

We report the crystal structures of EVE-HNH endonuclease VcaM4I in the absence of DNA, in complex with 5mC and 5hmC containing single-stranded DNA (ssDNA), and with 5mC containing double-stranded DNA (dsDNA). The structures show the binding mode of modified DNA to the EVE domain, and they help to identify the active site residues in the HNH domain. The crystallographically determined EVE domain pocket for a flipped modified cytosine and the assignment of active site residues are validated by testing VcaM4I variants for the *in vitro* activity on modified PCR DNA and ability to restrict T4gt or control phages in *E. coli* cells. Together with recent structural work on the 6mA sensing YTH domain of *Thermococcus gammatolerans* McrBC (18), our data contribute to better understanding of PUA superfamily domain containing restriction endonucleases.

## MATERIALS AND METHODS

### Expression and purification of full-length VcaM4I and its variants for biochemical assays

Synthetic genes for *vcaM4IR* restriction endonuclease (GenBank ID WP_010645282) and its variants were ordered from Integrated DNA Technologies (IDT) and cloned into pTXB1 plasmid by Hi-Fi assembly mix (NEB). The resulting constructs coded for fusion proteins with a C-terminal intein-chitin-binding domain (intein-CBD), which is cleavable in presence of thiol reagent. Expression constructs were transformed into *E. coli* T7 Express (C2566). Cells were grown to late log phase in LB medium supplemented with ampicillin. The cells were then supplemented with IPTG to a final concentration of 0.5 mM and kept at 18°C overnight. Wild type and mutant proteins were purified by chitin-affinity chromatography. Proteins were released from the captured C-terminal intein-CBD tag by overnight cleavage with DTT (50 mM). Concentrated proteins were kept in storage buffer containing 20 mM Tris–HCl (pH 7.5), 0.2 M NaCl, 10 mM DTT, 50% glycerol (Supplementary Figure S1). For EMSA experiments proteins were further purified by size exclusion chromatography using Superdex 200 10/300 GL followed by dilution in low salt buffer and purification with salt gradient on Hitrap Q HP ion exchange column. The protein was concentrated with simultaneous buffer exchange to 15 mM Tris–HCl, pH 8.5, 0.3 M NaCl and 1 mM TCEP, using Amicon^®^ Ultra-2 ml centrifugal filters.

### Expression and purification of VcaM4I HNH domain and its variants for biochemical studies and toxicity assay

VcaM4I fragments Helix-HNH (residues 148–309), HNH (residues 179–309) and HNH_H224A (residues 179–309) were soluble with N-terminal His-SUMO tag, but not with C-terminal intein-CBD tag. To assess toxicity, pET28 based expression constructs of the soluble variants, prepared in NEB Stable *E. coli* cells lacking λDE3 were transformed into *E. coli* expression strain BL21(λDE3), in the presence or absence of 1% glucose as an additional repressor of non-induced expression (from promoter leakage). For biochemical characterization, His-SUMO-Helix-HNH and His-SUMO-HNH_H224A were expressed in the BL21(λDE3) *E. coli* strain. The cells were grown to late log phase in LB medium supplemented with kanamycin. They were then supplemented with IPTG to a final concentration of 0.2 mM and kept at 16°C overnight. Cells were harvested by centrifugation at 4000 *g* for 20 min and stored at −20°C. The cell lysate was prepared by suspension of the pellet in a buffer containing 20 mM HEPES (pH 7.8), 5% glycerol, 0.5 M NaCl, 10 mM imidazole, pH 8.0, 1 mM PMSF. The proteins were purified by Ni-NTA affinity column chromatography. Elution was performed in buffer containing 20 mM HEPES, 5% glycerol, 0.2 M NaCl, 0.25 M imidazole (pH 8.0). Sumo protease was added to the elution fraction that was next dialyzed in order to remove imidazole from solution. The cleaved tag was removed by a second run through the Ni-NTA resin. Finally, the protein was purified and protein oligomeric state was analyzed on Superdex 75 10/300 GL column with simultaneous buffer exchange for 15 mM Tris–HCl (pH 8.5), 0.3 M NaCl and 1 mM TCEP. Bovine serum albumin, ovalbumin, myoglobin and vitamin B12 were used as molecular mass standards.

### Electrophoretic mobility shift assay

DNA binding properties of VcaM4I and its mutants were analyzed using dsDNAs with single Cy5 fluorescent label (Supplementary Table S1). Protein (50-1000 nM) was incubated with dsDNA (25 nM) for 60 minutes in binding buffer (15 mM Tris–HCl, pH 8.5, 160 mM NaCl and 2 mM DTT, 0.1 mM EDTA, 4.2% ficoll 400) at room temperature (∼23°C) followed by gel electrophoresis (1× TBE, 12% acrylamide 19:1 acrylamide:bisacrylamide). DNA and DNA–protein complex were visualized using the Biorad ChemiDoc™ MP Imaging System.

### *In vitro* activity assays

Unmodified, 5mC and 5hmC modified DNA fragments were prepared by PCR with a region of pBR322 as a template. dNTP mix with 5mCTP was provided by NEB. dNTP mix with 5hmCTP was purchased from Zymo Research. To exclude size effects on the rate of substrate degradation, two different mixes were prepared. In one mix, the lengths were 1.1 kb for unmodified DNA, 2.1 kb for 5mC-DNA, and 2.9 kb for 5hmC-DNA. In the second mix, lengths were reversed, to 2.9 kb for unmodified DNA, 2.1 kb for 5mC-DNA, and 1.1 kb for 5hmC-DNA. Comparison of digestion rates with both mixes showed that fragment length was unimportant for DNA cleavage rate, and that either mix could be used. Digestion experiments with VcaM4I and its variants were carried out in NEB Buffer 2.1 (50 mM NaCl, 10 mM Tris–HCl, 10 mM MgCl_2_, 0.1 mg/ml BSA, pH 7.9) for 30–60 min. The enzyme dilutions are indicated in figure legends, the DNA concentration was 0.5 μg (∼9 nM). After restriction digestion, proteinase K (1.6 U, from a stock of 800 U/ml, NEB) was added the to the digestion mixture and incubated at 37°C for 20 min to degrade DNA bound proteins. MDREs AbaSI (5 U), Eco15I (10 U) and PvuRts1I (20 U), and regular Type IIP enzyme HpaII (20 U) in NEB 2.1 buffer or CutSmart buffer (50 mM potassium acetate, 20 mM Tris-acetate, 10 mM magnesium acetate, 100 μg/ml BSA, pH 7.9), were used as controls. Short genomic DNA and RNA fragments were carried over from enzyme preparation and do not correspond to cleavage products.

### Qualitative *in vivo* restriction assays

For phage spot tests, log phase T7 Express (C2566) *E. coli* cells cultured in 0.4 ml phage broth (10 g/l soy peptone, 5 g/l NaCl, 0.5 g/l MgCl_2_, in distilled water, autoclaved) were mixed with 4 ml of soft agar and plated on Rich plate (10 g/l soy peptone, 5 g/l NaCl, 0.5 g/l MgCl_2_, 7.5 g/l agar, in distilled water, autoclaved) with ampicillin and 0.5 mM IPTG. After 10 min air drying at room temperature, diluted λvir (Dam^+^ Dcm^−^) and T4gt phages (6 μl) were spotted onto the cell lawn using repeat pipette. The plates were incubated overnight at 37°C. λvir phage dilutions equaled: 10^−4^, 10^-5^, 10^-6^, T4gt dilutions: 10^−5^, 10^−6^, 10^−7^. Alternatively, 8 μl of diluted λvir, T4 and T4gt phages were spotted onto cell lawns without IPTG. Phage dilutions equaled: λvir: 10^−3^, 10^−4^, 10^-5^, T4gt and T4: 10^−4^, 10^−5^, 10^−6^. T7 Express (C2566) cells expressing empty pTXB1 plasmid, and the plasmid containing *tagIR* gene were used as controls. λvir phage stock contained ∼1.1 × 10^9^ PFU/ml; T4gt and T4 stocks contained ∼5 × 10^9^ PFU/ml. For most variants the restriction experiment was performed in triplicate.

### Quantitative *in vivo* restriction assays

For phage plating assays, freshly diluted T4gt phage stocks (0.1 ml of 10^−6^ and 10^−7^ dilution) were used to infect log phase T7 Express (C2566) cells. After phage absorption for 15 min at room temperature, soft agar was added to the infected cells and plated on Rich agar plates supplemented with ampicillin (100 μg/ml) and IPTG (0.5 mM). Plates were incubated at 37°C overnight and phage plaques were scored as number of plaque forming units (PFU)/ml.

### Expression and purification of VcaM4I for crystallographic experiments

For large scale protein expression for crystallography, the original expression construct for VcaM4I was modified by addition of a hexa-histidine tag to the C-terminal intein-chitin fusion, so that affinity purification could be done using a Ni-NTA column (replacing the chitin beads). The tag was introduced by whole plasmid PCR using primers stated in Supplementary Table S1. VcaM4I was expressed in Rossetta (λDE3) *E. coli* strain in Studier's autoinduction media. Cells were harvested by centrifugation at 3200 *g* for 45 min and stored at −20°C. The cell lysate was prepared by suspension of the pellet in buffer R (40 mM Tris–HCl (pH 8.5), 5% glycerol, 0.5 M NaCl, 1 mM PMSF, 1 mM β-mercaptoethanol), sonication and centrifugation at 18 000 *g* and 4°C for 45 min. The supernatant was loaded on a HisTrap Crude column. Buffer W (20 mM Tris–HCl (pH 8.5), 5% glycerol, 0.5 M NaCl), buffer W with 1 M NaCl and buffer W with 25 mM imidazole were used for column washes at 5 column volumes each. Elution was carried out with buffer E (20 mM Tris–HCl (pH 8.5), 5% glycerol, 0.5 M NaCl, 0.2 M imidazole 8.0). The intein-CBD-His tag was cleaved by the addition of β-mercaptoethanol to 120 mM final concentration and 48 h incubation in 4°C. The buffer was exchanged for 20 mM Tris–HCl (pH 7.5), 5% glycerol, 0.5 M NaCl using HiPrep 26/10 desalting column. Subsequently, the sample was purified with HisTrap column that bound free tag and VcaM4I with uncleaved tag. Finally, the protein was purified on a Superdex 200 10/300 GL column with simultaneous buffer exchange for 15 mM Tris–HCl (pH 8.5), 0.3 M NaCl and 1 mM TCEP.

### Crystallization

VcaM4I and its complexes were crystallized using hanging drop vapor diffusion method at room temperature. In all experiments, protein or protein/DNA solution was mixed with reservoir buffer in 1:1 ratio.

VcaM4I alone was crystallized from a 7.2 mg/ml solution in 0.27 M NaCl, 13.5 mM Tris–HCl (pH 8.5), 0.2 M sodium malonate and 0.9 mM TCEP. This solution was mixed with and equilibrated against reservoir buffer containing 1.8 M ammonium sulfate, 0.1 M MES (pH 5.25). The Hg^2+^ derivative for phasing was prepared by a 45 min crystal soak in 20 mM thiomersal dissolved in reservoir solution. Crystals were cryoprotected in perfluoropolyether oil.

VcaM4I with ssDNA or dsDNA was crystallized from a solution containing 8 mg/ml of protein and a 1.15:1 molar excess of ssDNA or dsDNA over protein (subunit, not dimer) in 0.3 M NaCl, 15 mM Tris–HCl (pH 8.5) and 1 mM TCEP. The protein was incubated with DNA on ice for 30 min, and then mixed with reservoir solution containing 0.1 M MES (pH 5.25) and 1.6 M ammonium sulfate for ssDNA complexes and 1.2 M ammonium sulfate for dsDNA complex. For the complex of VcaM4I with 5hmC containing ssDNA, 0.1 M spermine tetrahydrochloride was additionally used, but the morphology of the crystals was not affected by the presence of the additive. Reservoir solutions were diluted with glycerol to a final concentration of 30% for cryoprotection.

### Structure determination

VcaM4I in the absence of DNA formed orthorhombic crystals that contained a protein dimer in the asymmetric unit. Crystals diffracted to 1.50 Å at the EMBL/DESY P13 beamline and were assigned to space group *P*2_1_2_1_2_1_ based on the extinctions of odd-index reflections on all three reciprocal axes. The phase problem was solved by single anomalous diffraction, for a soak with Hg^2+^ ions. Harker sections at *x* = 1/2, *y* = 1/2 and *z* = 1/2 featured strong peaks, clearly indicating that the soaking procedure had been successful. The heavy atom structure was interpreted using the HKL2MAP (20) wrapper for the SHELX suite (21). Phasing and initial model building in SHELXE (21) demonstrated a clear contrast between the two enantiomeric heavy atom structure alternatives. The phases from SHELXE were then used to build an almost complete model of VcaM4I in ARP/wARP (22). The crystals of VcaM4I in complex with single-stranded CA5hmCAG and CA5mCAG oligonucleotides grew in *P*6_2_22 space group and diffracted to 1.55 and 1.48 Å at the EMBL/DESY P14 beamline, respectively. A single protomer of VcaM4I was used to solve the structure of the VcaM4I-5hmC_ssDNA complex with the help of the PHASER program (23). The single-stranded oligonucleotide was built manually in COOT (24). The refined structure was directly mapped into the VcaM4I-5mC_ssDNA complex crystal of almost identical unit cell dimensions. The crystals of VcaM4I in complex with blunt-ended, hemimethylated dsDNA of the CCATG5mCGCTGA/TCAGCGCATGG sequence grew in *P*6_1_22 space group and diffracted only to 3.14 Å. The X-ray data for this weakly diffracting crystal form were truncated with permissive CC_1/2_ (25) 30% cut-off. The structure was solved with the help of the PHASER program (23) and the model of the VcaM4I dimer. The DNA was built manually, refined and adjusted to form canonical Watson-Crick base pairing except for the central base pair and some outmost pairs of the oligoduplex that were partly disordered. The structures were refined with the help of REFMAC (26), PHENIX (27) and COOT (24) programs. The data collection and refinement statistics are presented in Table 1. The final model coordinates and the corresponding structure factors were deposited in the Protein Data Bank with the following accession codes: 6YEX (VcaM4I in the absence of DNA), 6YJB (VcaM4I-5hmC_ssDNA complex) 6YKF (VcaM4I-5mC_ssDNA complex) and 6YMG (VcaM4I-dsDNA complex).

**Table 1.** Data collection and refinement statistics

	VcaM4I in the absence of DNA	VcaM4I-5hmC-ssDNA complex	VcaM4I-5mC-ssDNA complex	VcaM4I-5mC-dsDNA complex
**DATA COLLECTION**				
Space group	*P*2_1_2_1_2_1_	*P*6_2_22	*P*6_2_22	*P*6_1_22
Cell dimensions				
*a* (Å)	57.0	128.7	129.0	81.1
*b* (Å)	93.5	128.7	129.0	81.1
*c* (Å)	126.3	110.9	110.9	617.3
Wavelength (Å)	1.0064	1.0064	0.97626	0.9184
Resolution (Å)	**1.50**	**1.55**	**1.48**	**3.14**
lowest shell	(30–4.45)	(30–4.60)	(50–4.41)	(50–9.28)
highest shell	(1.59–1.50)	(1.64–1.55)	(1.56–1.48)	(3.33–3.14)
*R*_meas_ (%)^a^	**4.6** (2.8, 110.4)	**10.3** (3.6, 236.4)	**9.6** (4.3, 101.9)	**22.7** (6.8, 164.1)
CC_1/2_ (%)^a^	**100** (99.9, 85.1)	**100** (100, 75.2)	**99.9** (99.9, 74.2)	**99.1** (99.9, 30.6)
I/σI^a^	**26.1** (82.7, 2.27)	**27.0** (107.4, 1.96)	**15.0** (46.2, 1.91)	**8.6** (34.5, 0.89)
Completeness (%)^a^	**99.2** (99.1, 98.2)	**99.9** (99.2, 99.7)	**98.9** (95.1, 98.5)	**92.8** (98.6, 74.4)
Multiplicity^a^	**13.1** (11.5, 13.4)	**39.2** (34.3, 39.9)	**10.6** (9.8, 10.1)	**9.6** (13.6, 5.2)
No. reflections	107966	78576	90208	20874
**REFINEMENT**				
*R*_work_	19.33	14.25	16.93	24.15
*R*_free_^b^	21.32	16.36	18.48	26.62
No. atoms^c^	6509	3610	3659	6063
Protein	5437	2803	2849	5039
DNA	0	101	100	892
Solvent	1072	706	710	132
Bond lengths rmsd (Å)	0.006	0.008	0.006	0.003
Bond angles rmsd (°)	1.13	1.36	1.29	1.19
Ramachandran				
allowed (%)	100	100	100	100
favored (%)	97.7	98.7	98.7	98.7
MolProbity clashscore	1.5	2.4	1.7	1.84
PDB code	6YEX	6YJB	6YKF	6YMG

^a^Lowest and highest shell in brackets.

^b^5% of reflections were set aside randomly.

^c^Double conformations counted separately.

### Small angle X-ray scattering experiments

Double-stranded DNA oligoduplexes of three different lengths containing variably spaced 5hmC residues (Supplementary Table S1) were suspended in buffer containing 30 mM Tris-HCl, pH 7.2, 150 mM NaCl, 2 mM TCEP, 5% glycerol, 1 mM EDTA and annealed in thermocycler. The short oligoduplex analogous to the one used for crystallization was in 20 mM Tris–HCl, pH 8.5. The oligos were mixed equimolarly with VcaM4I protein and dialyzed against the former buffer using Slide-A-Lyzer™ MINI Dialysis Device 3.5K MWCO. Dilution series was prepared on site by local contact with provided buffer. Small angle X-ray scattering data for VcaM4I alone and in complex with dsDNA fragments listed above were collected at the EMBL/DESY P12 beamline. The data collection was performed at 20°C on a Pilatus 6M (Dectris) detector with at 10 keV photon energy (corresponding to a wavelength ∼0.1240 nm), with 0.05 s exposure per frame. Diffraction intensities were recorded to a maximum Bragg angle of 10.94°, but only momentum transfers 4π sin θ/λ (in natural units with *h*/2π = 1) up to 5/nm were used for the comparison of theoretical and experimental scattering curves. In all cases, signal from matched buffer was subtracted, and several protein or protein DNA concentrations were analyzed, in several repeats. Only datasets with most frames retained in the analysis that were not classified as over-subtracted were analyzed further. The CRYSOL program (28) was used to calculate predicted scattering curves. Agreement of predicted scattering curves for mixtures with experimental data was assessed using the OLIGOMER program (29).

## RESULTS

### VcaM4I crystal structures

**Figure 1.**
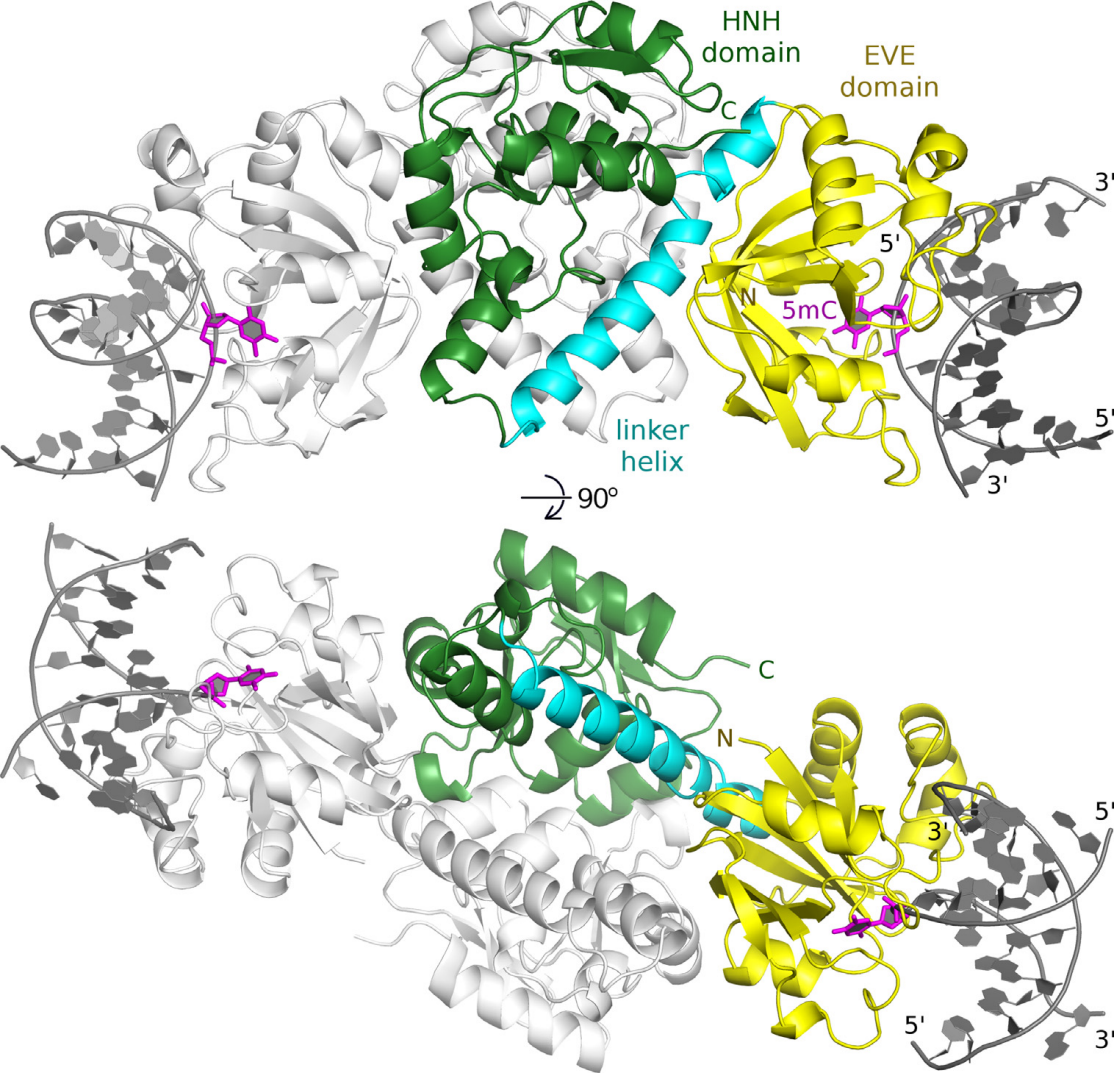
Overall structure of the VcaM4I–dsDNA complex. One protomer of the VcaM4I dimer is colored: the N-terminal EVE domain (residues 1–147) is in yellow, the helical linker (residues 148–178) in cyan, and the C-terminal HNH domain (residues 179–309) in green. The other protomer and the two dsDNA molecules bound to the EVE domains are in gray. The dimer axis runs vertically in the top panel and towards the reader in the bottom one. The 5-methylcytosine residues flipped out of the DNA stack are indicated in magenta.

VcaM4I was expressed with good yield as an intein fusion to a histidine-tagged chitin-binding domain (CBD), purified from extracts by affinity chromatography, and liberated from the tags by thiol agent triggered intein cleavage. After re-buffering by gel-filtration, the protein could be crystallized on its own, in complex with short ssDNA containing 5mC or 5hmC, and with dsDNA containing 5mC in one strand. Except for the weaker diffracting crystals of VcaM4I with dsDNA, the resolution of the data reached around 1.5 Å. The crystal form without DNA was phased experimentally. The other structures were solved by molecular replacement (Table 1). Although crystals without DNA, with ssDNA, and with dsDNA grew in different space groups, the protein conformation was remarkably similar in all structures, with the exception of two loops in proximity of the DNA (residues 24–34 and 72–84). Therefore, the structures can be described together (Figure 1 and Supplementary Figure S2).

In all crystal structures, VcaM4I protomers have the predicted two domain architecture, with N-terminal domains of the PUA superfamily (residues 1–147), linker helices (148–178), and C-terminal HNH domains (residues 179–309). In all crystal forms, the protomers dimerize via the HNH domains. The extensive interface area of 2000 Å^2^ (equivalent to 4000 Å^2^ of buried surface) and the classification of the interface as stable by the PDBePISA server (30) suggest that the dimerization mode in the crystals is relevant in solution. This conclusion is further strengthened by a comparison of the VcaM4I HNH domain dimerization mode with the dimerization mode observed in other structurally characterized HNH endonucleases (Supplementary Figure S3). The C-terminal HNH domains anchor the N-terminal domains via long α-helical linkers, and extensive non-covalent contacts. As a consequence of this anchoring, the N-terminal domains also follow the (local or crystallographic) two-fold symmetry that relates the HNH domains. The ssDNA and dsDNA molecules in the crystals are bound to the N-terminal domains. The DNA binding site of the C-terminal HNH domains is consistently empty in all structures.

### The VcaM4I N-terminal domain belongs to the EVE family

The bioinformatic analysis assigned the N-terminal domain of VcaM4I to the PUA-superfamily (19). At present, two partially conflicting systems for sub-classification are in use (Supplementary Figure S4).

**Figure 2.**
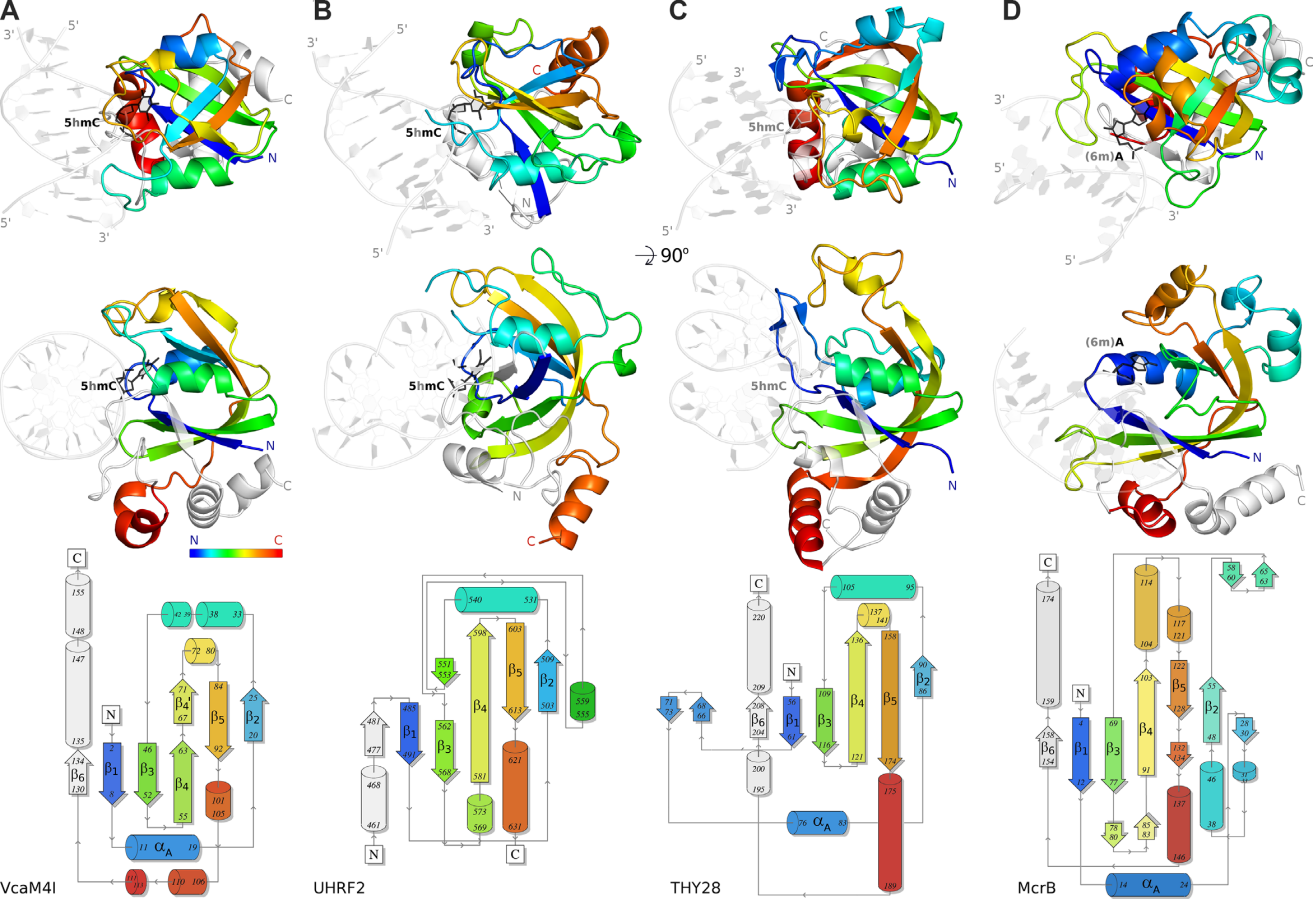
Structure of the N-terminal EVE domain of VcaM4I in comparison to other PUA-superfamily domains. The figure compares (**A**) the N-terminal EVE domain of VcaM4I with (**B**) the SRA domain of UHRF2 (PDB ID 4pw5 (36)), (**C**) the EVE domain of THY28 (PDB 5j3e, unpublished), (**D**) and the 6-methyladenine binding YTH domain of McrB from *T. gammatolerans* (PDB 6p0g (18)). Top: cartoon representation of the domain structures colored from N- to C-terminus (blue to red) in two orientations. The N- and C- terminal fragments outside of the core 5-stranded β-sheet are colored in light gray. The dsDNA complexes of VcaM4I, UHRF2 and McrB were determined experimentally. The complex of THY28 is a model based on the 5j3e coordinates, altered by flipping of a single base and substituting it with 5hmC. DNA polarity was consistent except in the McrB–DNA complex. A revised model presented in the figure with consistent DNA strand orientation is at least as compatible with the diffraction data as the published model. Bottom: topology diagrams of the domains generated with the PDBSUM program (47), with minor manual adjustments necessary for direct comparison with the structures. The core secondary structure elements of the domains are labelled.

The qualitative classification system of Bertonati and colleagues (16) identifies five-stranded, mixed β-sheet as the core motif of PUA superfamily domains. The presence or absence of an additional β-strand adjacent to β1 of the core motif, and its location in the sequence, decide the sub-classification as EVE, ASCH, YTH or a genuine PUA domain (in the strict sense). The N-terminal domain of VcaM4I has the defining five-strand motif (strands β1 to β5) of the PUA superfamily and an additional β-strand (labelled β6) adjacent to β1, downstream of the core motif in the sequence (Figure 2A). This classifies the domain as an EVE domain according to the qualitative criteria.

In databases, such as InterPro (31), Pfam (32) and PDB (33), the sub-classification of PUA superfamily domains is based on quantitative criteria. According to DALI, the proteins with highest similarity to the N-terminal domain of VcaM4I all belong to the EVE family (top *Z*-score 14.3, i.e. similarity is 14.3 standard deviations above the mean). The list of high-scoring EVE domains includes bacterial PSPTO5229 from *Pseudomonas syringae*, whose structure led to the recognition that EVE domains belong to the PUA superfamily (16), and human thymocyte nuclear protein 1 (THYN1 or THY28), an EVE domain protein (34) enriched in a screen for 5hmC binding proteins (17). YTH domains, generally associated with 6-methyladenosine recognition, featured below EVE domain proteins, but were also prominently represented in the list. Other related domains of the PUA superfamily, including primarily ASCH domains, were classified as more distant (Supplementary Table S2 and Supplementary Figure S5).

Hence, the qualitative and quantitative PUA superfamily assignment schemes both support the classification of VcaM4I as an EVE domain. As the domain is more distant to previously known EVE domains than these are to each other according to CLANS (35) analysis, our characterization of the N-terminal domain of VcaM4I substantially extends the scope of this protein family.

### The N-terminal EVE domain of VcaM4I flips the modified base

The EVE domain of VcaM4I binds DNA on the equivalent face and in very similar orientation as SRA domains (Figure 2A, B). Qualitatively, the VcaM4I DNA binding mode is also similar to the one observed in the co-crystal structure of the THY28 EVE domain with 5mC containing dsDNA (with 5mC in an irrelevant position, PDB ID 5j3e) (Figure 2C) and to the recently published YTH domain featuring McrB of *T. gammatolerans* (18) (Figure 2D).

**Figure 3.**
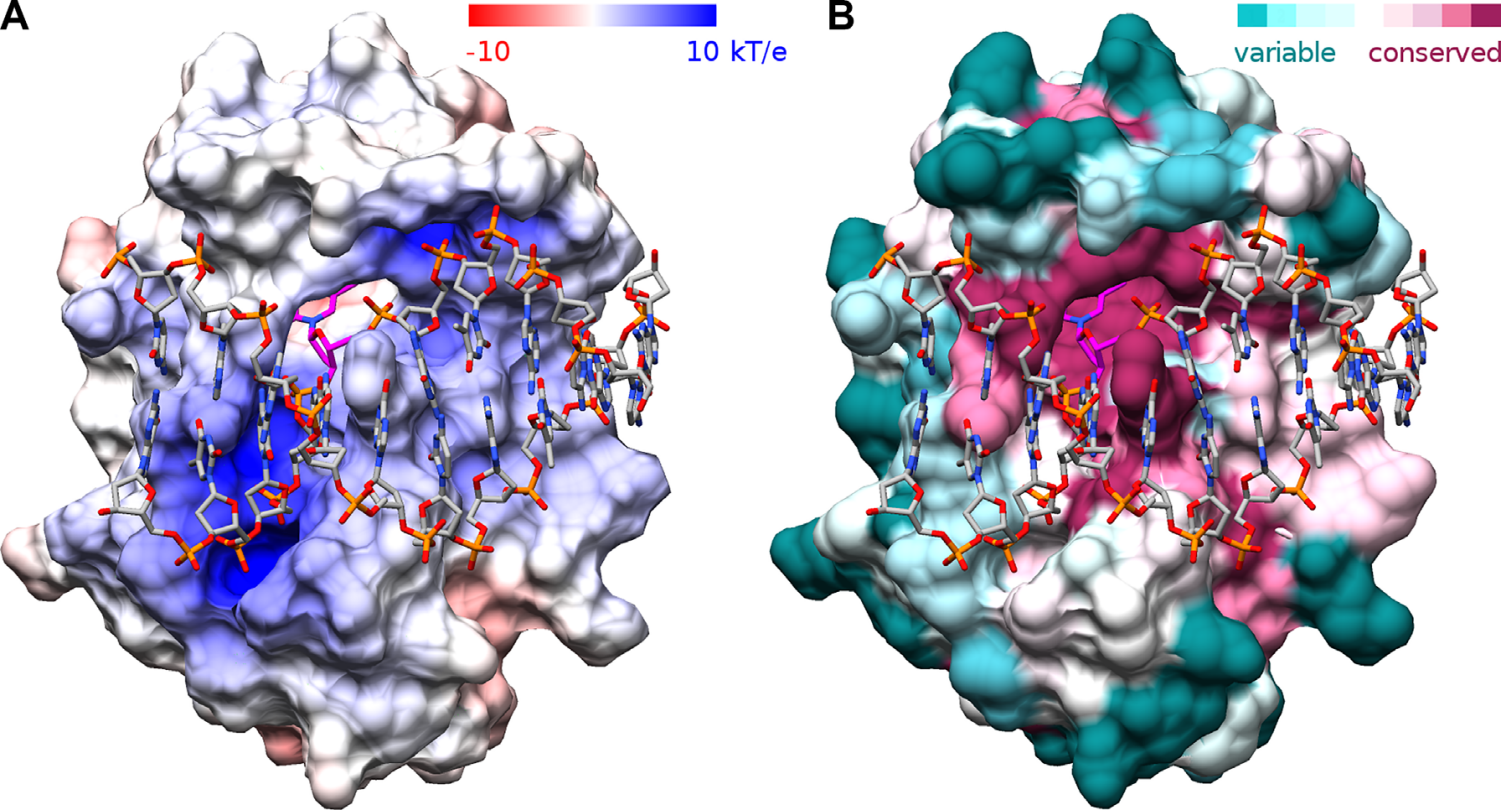
DNA binding to the VcaM4I EVE domain. (**A**) Electrostatic potential was generated with APBS (48) and mapped to the VcaM4I surface with CHIMERA (49). Negatively charged surface regions are red, positively charged ones blue. (**B**) Amino acid conservation scores for the EVE domain were generated with ConSurf with default parameters (50) and mapped with CHIMERA (49). The highest conservation is in the region of the flipped base. The wedge intercalates into dsDNA with Q128 residue, the pocket accommodates the flipped modified base (shown in magenta).

**Figure 4.**
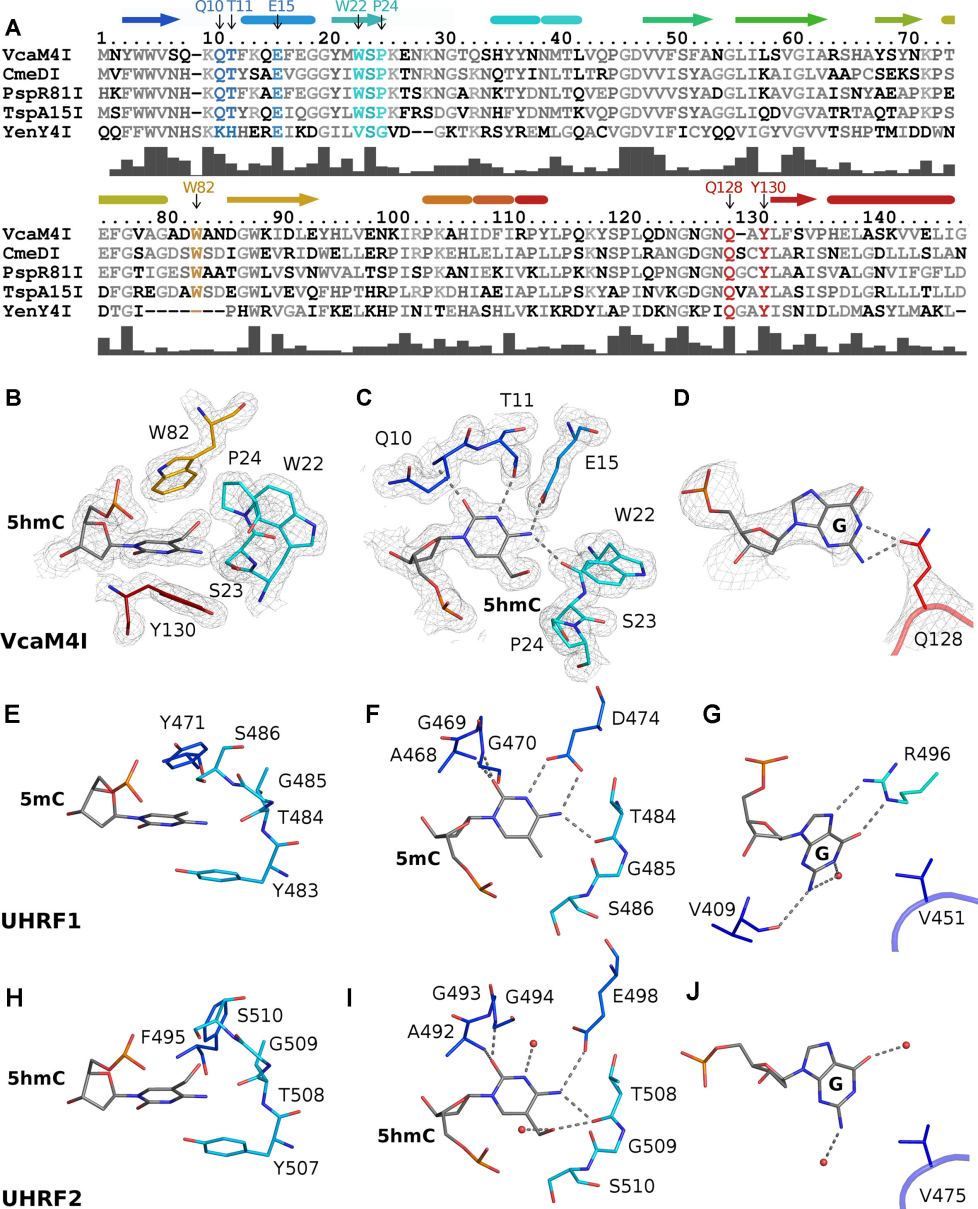
Specific interactions of the VcaM4I EVE domain with DNA. (**A**) Alignment of EVE domains of modification dependent restriction endonucleases. The bars under the alignment indicate the degree of sequence conservation. (**B**) ‘Side’ view of the VcaM4I EVE pocket demonstrating the sandwiching of the flipped modified base. (**C**) ‘Top’ view of the VcaM4I modified base binding pocket, showing the specific interactions of its Watson-Crick edge with the protein. (**D**) Interactions of the estranged base with Q128 of the VcaM4I EVE domain. The model and the composite omit electron density map are based on the 5hmC ssDNA complex for panels B-C (1.5 rmsd contour level) and on dsDNA complex for panel D (1.3 rmsd contour level). (**E–G**) Equivalent views of the interactions of the SRA domain of UHRF1 with 5mC containing DNA (8). (**H–J**) Equivalent views of the interactions of the SRA domain of UHRF2 with 5hmC containing DNA (36). Color coding of secondary structure elements (helices represented by cylinders, β-strands by arrows) and functional residues is consistent with Figure 2 (the N- and C-termini were included in the rainbow-colored region to indicate their sequence locations).

The DNA binding face of the VcaM4I EVE domain is positively charged (Figure 3A). It features a wedge and a hydrophobic pocket, which are among the most conserved regions on this face of the domain (Figures 3B and 4A, Supplementary Figures S6 and S7). The pocket accommodates the flipped 5mC or 5hmC base in all co-crystal structures. The faces of the nucleobase stack against Y130 on one side and W82 (plus to a lesser extent P24) on the other (Figure 4B). The Watson–Crick edge of the base (i.e. the edge that would be involved in base pairing if the base was not flipped) is engaged in extensive hydrogen bonding interactions. Its exocylic O2 and endocylic N3 atoms accept hydrogen bonds from the main chain amide of Q10 and hydroxyl group of T11, and the N4 atom donates hydrogen bonds to the main chain carbonyl of W22 and the Oϵ of E15 (Figure 4C). In the DNA stack, Q128 fills, at least in part, the space normally taken by the flipped base. The side chain substitutes for the base by van der Waals stacking, and also accepts hydrogen bonds from the N1 and N2 nitrogen atoms of the estranged guanine that was paired with 5mC or 5hmC prior to flipping (Figure 4D). Functionally, the EVE domain of VcaM4I is similar to the SRA domain of UHRF2 in its preference for 5hmC over 5mC (19,36). However, the pocket of the EVE domain of VcaM4I is more hydrophobic than the pocket of the SRA domain of UHRF2 (Figure 4H,I), and thus more similar to the more hydrophobic pocket of UHRF1 (8,9) (Figure 4E,F). Interactions with the estranged guanine base also differ between EVE and SRA domains (Figure 4D,G,J).

### Biochemical characterization of the EVE domain variants

To biochemically confirm the relevance of residues forming the pocket for the modified base, we created variants of VcaM4I with altered EVE domain pockets. In a first round of experiments, we replaced various amino acids in the pocket or the intercalating Q128 by alanine residues. This set of variants was then assayed by electrophoretic mobility shift assay (EMSA), by *in vitro* digestion of a mix of unmodified, 5mC or 5hmC containing DNA, and by a phage spot assay using T4gt phage (with 5hmC instead of C in genomic DNA). In the EMSA assays, the wild-type protein displayed a preference for 5mC or 5hmC containing DNA over non-modified DNA, without much discrimination between the modified oligoduplexes (Supplementary Figure S8). Variants were therefore only assayed with non-modified and 5mC containing DNA. Many of them showed a reduced capacity to distinguish unmodified from 5mC containing DNA. In some cases, such binding was similar to the interaction of the wild-type enzyme with modified DNA, in the other cases, it resembled binding of the wild-type protein to non-modified DNA. We interpret this result as evidence that changes to the pocket walls or the intercalating residue affect the VcaM4I ability to distinguish modified and non-modified DNA, but also to modulate the overall non-specific affinity to DNA (Supplementary Figure S9).

VcaM4I was more active in *in vitro* digestion assays in the presence of Mn^2+^ than Mg^2+^ ions (Supplementary Figure S10, (19)). Moreover, it digested 5hmC containing DNA faster than 5mC containing DNA, despite the lack of a clear preference for 5hmC over 5mC in the EMSA assay. Among the VcaM4I variants, only E15A and Y130A had clearly impaired activity towards 5hmC containing DNA (Supplementary Figure S11). In a qualitative phage spot assay, propagation of phage T4gt was not noticeably restricted by the W22A variant, which carried an unintended additional frameshift mutation and could therefore serve as a control. Phage T4gt propagation was also not restricted by the Y130A variant which was among the two variants with clearly impaired *in vitro* nuclease activity. All other variants retained some restriction activity towards phage T4gt (but not the control phage λvir) that was more pronounced in overexpression than basal expression conditions. Thus, in the original set, Y130A was the only VcaM4I variant that was clearly impaired in all three assays (Supplementary Figures S12–S14, Supplementary Table S3). We conclude from this set of experiments that the EVE domain is very tolerant of subtractive amino acid exchanges (replacing a larger side chain by an alanine methyl group), mirroring in this respect the properties of the phylogenetically unrelated NEco domain (12).

**Figure 5.**
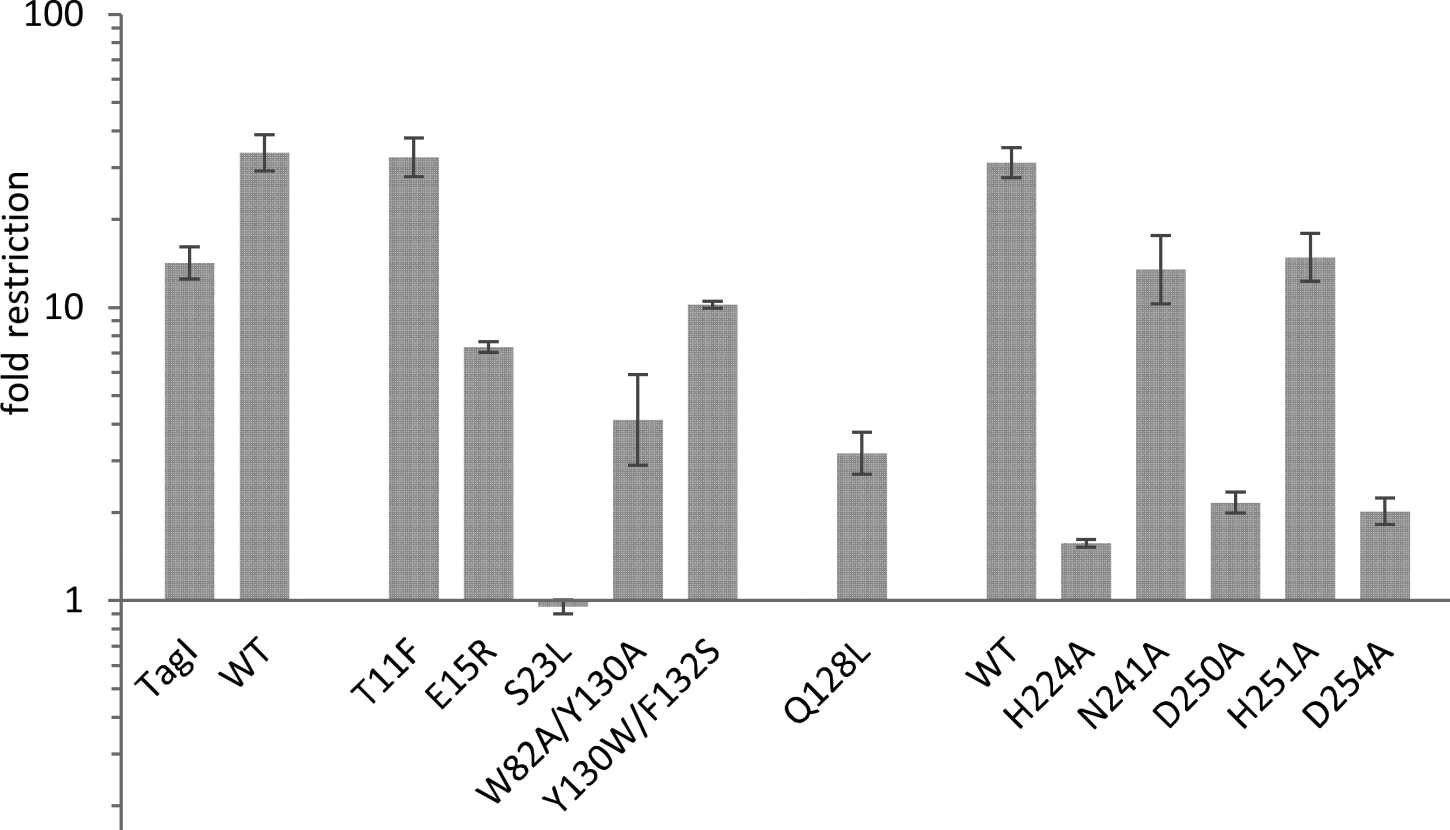
Phage T4gt plaque forming unit (PFU) assay on cells carrying wild-type and mutant VcaM4I expressed in the presence of IPTG. Cells carrying Q10V, W82R and Y130W variants formed poor cell lawns (mutants are toxic to the host in the presence of IPTG) and the three mutants could not be tested for the *in vivo* restriction activity.

As the originally designed VcaM4I variants (with the exception of the Y130A variant) did not behave as differently from the wild-type enzyme as expected, we carried out a second round of site-directed mutagenesis. This time, we designed either double exchanges, or substitutions to amino acids other than alanine. The variants were subjected to the *in vitro* digestion and phage restriction assays. In the *in vitro* activity tests, S23L and the double exchange W82A/Y130A and Y130W/F132S VcaM4I variants had drastically impaired activity both in the presence of Mg^2+^ and Mn^2+^ ions (Supplementary Figure S15). For *in vivo* assay, the phage spot test was replaced by a more quantifiable phage plating assay. The wild-type VcaM4I was more effective in restriction than the control restriction endonuclease TagI (3) (approx. 34-fold and 14-fold plaque count reductions, respectively). Restriction activity was completely abolished for the S23L variant. All other VcaM4I variants retained statistically significant restriction activity above background (one sided *t*-test, *P*-values < 0.05). However, with the exception of the T11F variant, the activity of the variants was significantly reduced compared to the wild-type (one sided *t*-test, *P*-values < 0.05) (Figure 5). Partial retention of restriction activity of the variants was not surprising. In a previous study of EcoKMcrA (a restriction endonuclease with phylogenetically unrelated 5mC/5hmC sensor domain), a similar robustness of restriction with respect to alterations of the pocket wall forming residues was also observed (12). We conclude from these experiments that the crystallographically determined binding mode of modified DNA to the VcaM4I EVE domain is relevant also in solution.

### The C-terminal HNH domain of VcaM4I contains the canonical ββα motif

**Figure 6.**
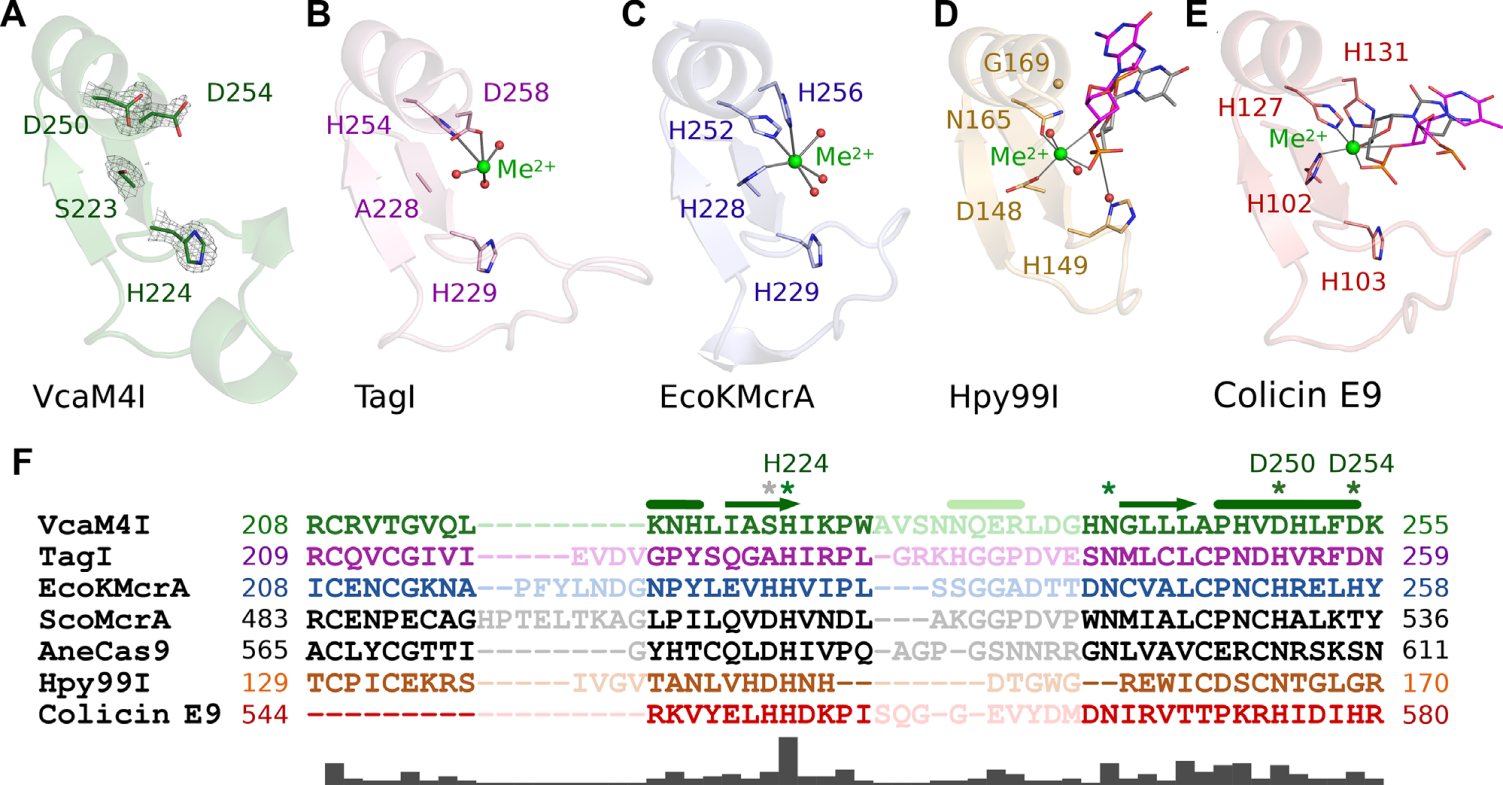
ββα-Me core of the VcaM4I HNH domain in comparison with related endonucleases. Top row presents the ββα-Me regions of (**A**) VcaM4I, (**B**) TagI (PDB 6ghs, (3)), (**C**) EcoKMcrA (PDB 6ghc (4)), (**D**) Hpy99I (PDB 3fc3 (38)) and (**E**) colicin E9 (PDB 1v15 (40)). The composite omit density map in (A) was based on the structure of VcaM4I with 5hmC-modified ssDNA and contoured at 1.5 rmsd. The coordination of the metal ions is indicated with faint lines. Additional faint line in (D) indicates the distance between the potential nucleophilic water and the scissile phosphate. The H103A mutation present in the structure used for (E) was *in silico* mutated back to the active site histidine but a catalytically unproductive conformer was chosen. (**F**) Structure-based sequence alignment of VcaM4I and similar HNH domains. The alignment was corrected manually. The faint regions indicate the lack of direct structural correspondence. The active site residues are marked with asterisk. Gray asterisk indicates the metal ligand present in some endonucleases which lost its function in VcaM4I and TagI. The bars under the alignment indicate the degree of sequence conservation.

Based on the prior work, the C-terminal domain of VcaM4I was classified as an HNH or ββα-Me domain (19). The hallmark ββα motif (37), built from two anti-parallel β-strands and an α-helix is indeed present in the enzyme nuclease domain (Figure 6A). Overall, the motif is similar to the core motif in other HNH endonucleases, such as TagI, EcoKMcrA, Hpy99I or colicin E9 (Figure 6B–E). In the catalytic domain of VcaM4I and the other proteins, the loop between the two β-strands is uncharacteristically elaborate for a hairpin, and the connection from the second strand of the hairpin to the α-helix is fairly direct.

Endonuclease HNH domains contain a single metal cation in the active site. They are named for three moniker residues, a histidine (H, activator of the attacking water), asparagine (N, not involved in catalysis, conserved for structural reasons), and another histidine (H, metal cation ligand). According to the sequence comparison and structural superposition, the relevant residues in VcaM4I are H224, N241 and D250. The replacement of moniker residues by their functional equivalents, as in the case of D250 playing a role of a histidine, is not uncommon (38,39) (Figure 6F). In many HNH endonucleases, the residue immediately preceding the first histidine of the HNH motif is a metal cation ligand. However, in VcaM4I, this residue is a serine, which is not suitable for metal ion coordination. Instead, spatial position suggests that in VcaM4I D254 could be its functional substitute.

In order to confirm the assignment of active site residues, we replaced them separately by alanine residues. As anticipated, the H224A, D250A and D254A variants drastically impaired restriction endonuclease activity *in vitro* (Supplementary Figure S16). Next, we tested VcaM4I variants in the phage plating assay, in protein overexpression conditions. All variants were significantly impaired in their restriction activity (one sided *t*-test, *P*-values < 0.05). Exchanges of N241, believed to play a structural role, and of H251, without clear role in catalysis, had only very mild effects on restriction activity. By contrast, the reduction was much more drastic for the H224A, D250A and D254A variants. However, even these variants retained statistically significant activity (one sided t-test, *P*-values < 0.05) (Figure 5). The variants retained some activity also in the phage spot assay, but only under induction conditions (Supplementary Figures S12 and S14). Partial retention of restriction activity of catalytically dead restriction endonuclease variants in overexpression conditions is not surprising, and has been reported before, for example in case of EcoKMcrA (12).

### A model for DNA bound to the catalytic domain and auto-inhibition by the linker helix

**Figure 7.**
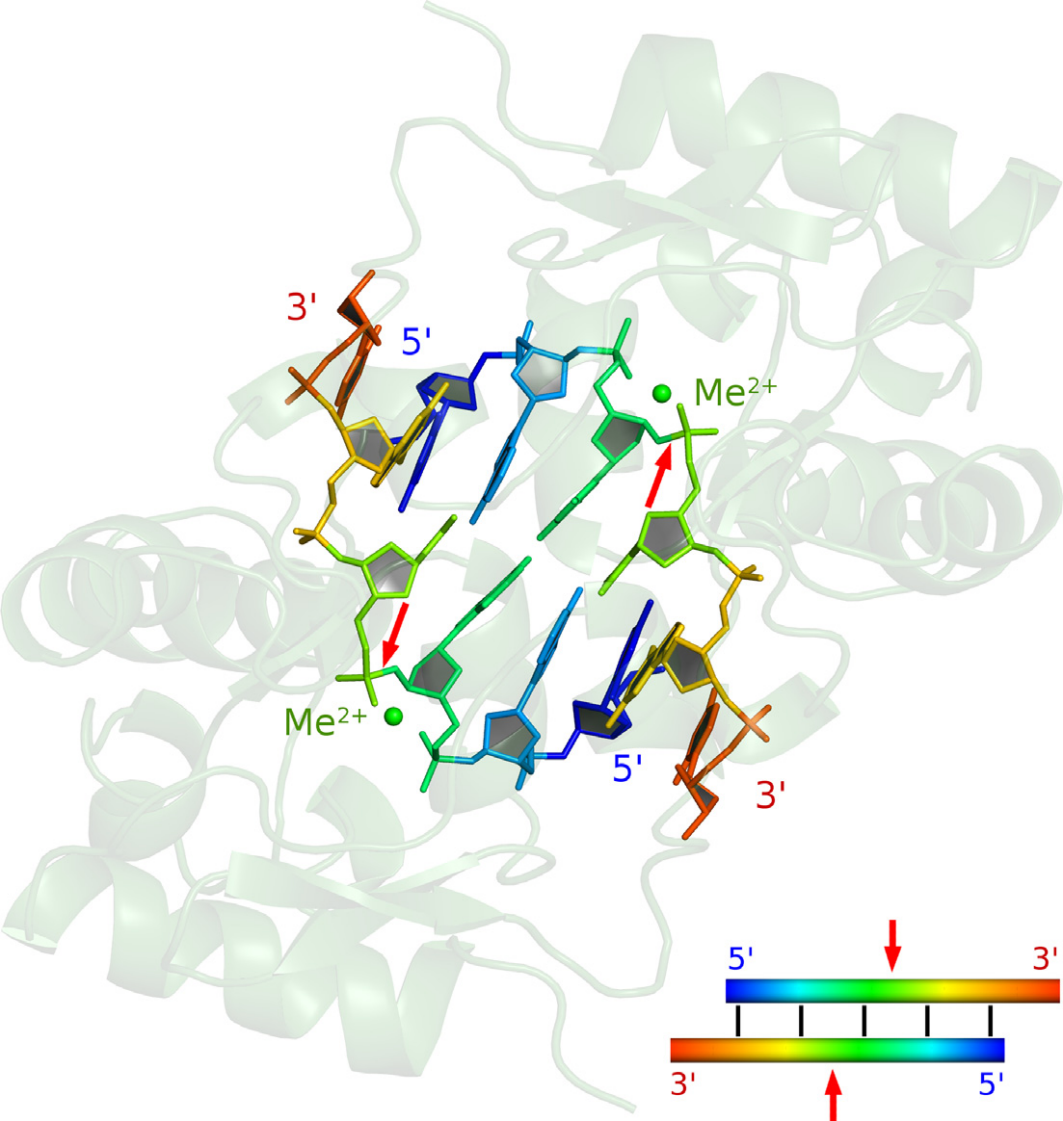
Model of the VcaM4I HNH domain dimer bound to DNA. The model is based on the co-crystal structure of colicin E9 with DNA (40). For each subunit, the substrate strand was modelled separately based on a superposition of the core catalytic ββα-motif in VcaM4I and colicin E9. Although this was not imposed by the modelling, the two strands base-pair (with Watson-Crick hydrogen bond distances shorter than expected). The red arrows indicate the sites of cleavage. The model is consistent with the experimental observation that VcaM4I generates fragments with single nucleotide 3′-overhangs.

As we were not able to obtain a co-crystal structure of VcaM4I in complex with DNA bound to the HNH domains, we resorted to modeling. The dimer of HNH domains alone is crescent-shaped and could accommodate dsDNA (∼20 Å in diameter). The dimer symmetry axis coincides with one of the DNA pseudo-2-fold axes. In the region of the ββα-Me motif, VcaM4I is highly similar to colicin E9. We therefore used a co-crystal structure of this protein with DNA (40) as our modeling template. For each VcaM4I protomer, only the ‘proximal’ substrate strand was modeled. Hence, the two strands need not form Watson–Crick pairs. However, except for a slightly close contact between DNA bases, a regular double helix was nearly regenerated, indicating that dsDNA can be accommodated with very slight conformational adjustments. As the bases from the two strands pair and as it is known which phosphodiester bonds are cleaved by the ββα-Me motif, the stagger of double-strand cleavage by VcaM4I can be deduced from the model. Inspection shows that single nucleotide 3′-overhangs are expected, exactly as experimentally observed (Figure 7). Agreement of the experimental and predicted double-strand break stagger and the pronounced positive charge of this face of the protein, also in comparison with other HNH endonucleases, are strong support for the validity of the modelling (Supplementary Figure S17).

**Figure 8.**
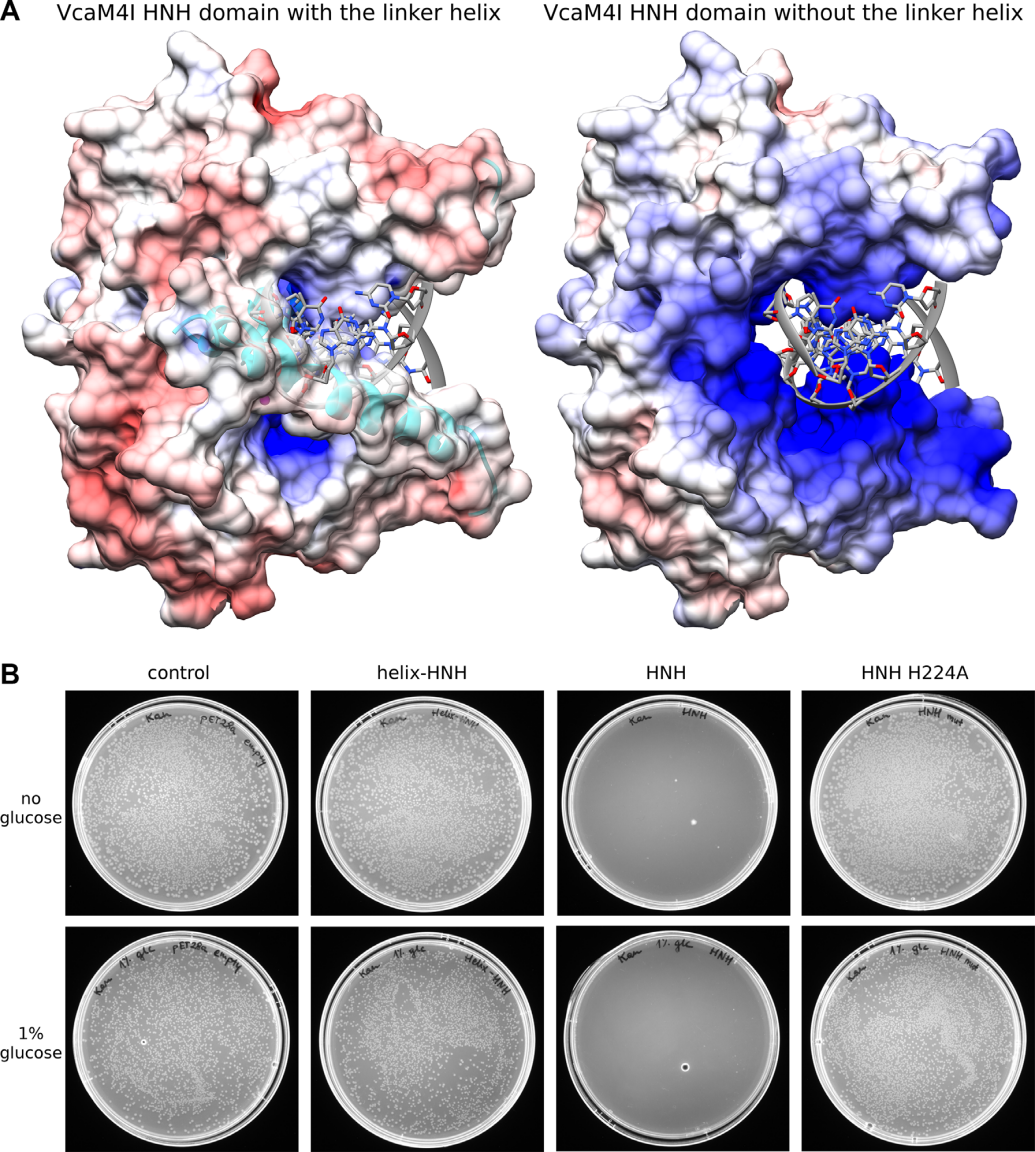
VcaM4I catalytic activity is regulated by the presence of the linker helix. (**A**) The depiction of the surface of the VcaM4I HNH domain dimer in the presence and absence of the linker helix. The DNA was not present in the structure but modelled as described in Figure 7. The electrostatic potential was calculated by APBS (48) with modelled Mg^2+^ ions in the active sites and mapped on the domain dimer surface with CHIMERA (49). (**B**) The VcaM4I HNH domain with and without the linker helix, as well as its catalytic mutant were expressed in *E. coli*. The domain without the helix and the inactivating mutation is toxic to the cells due to nonspecific endonuclease activity.

**Figure 9.**
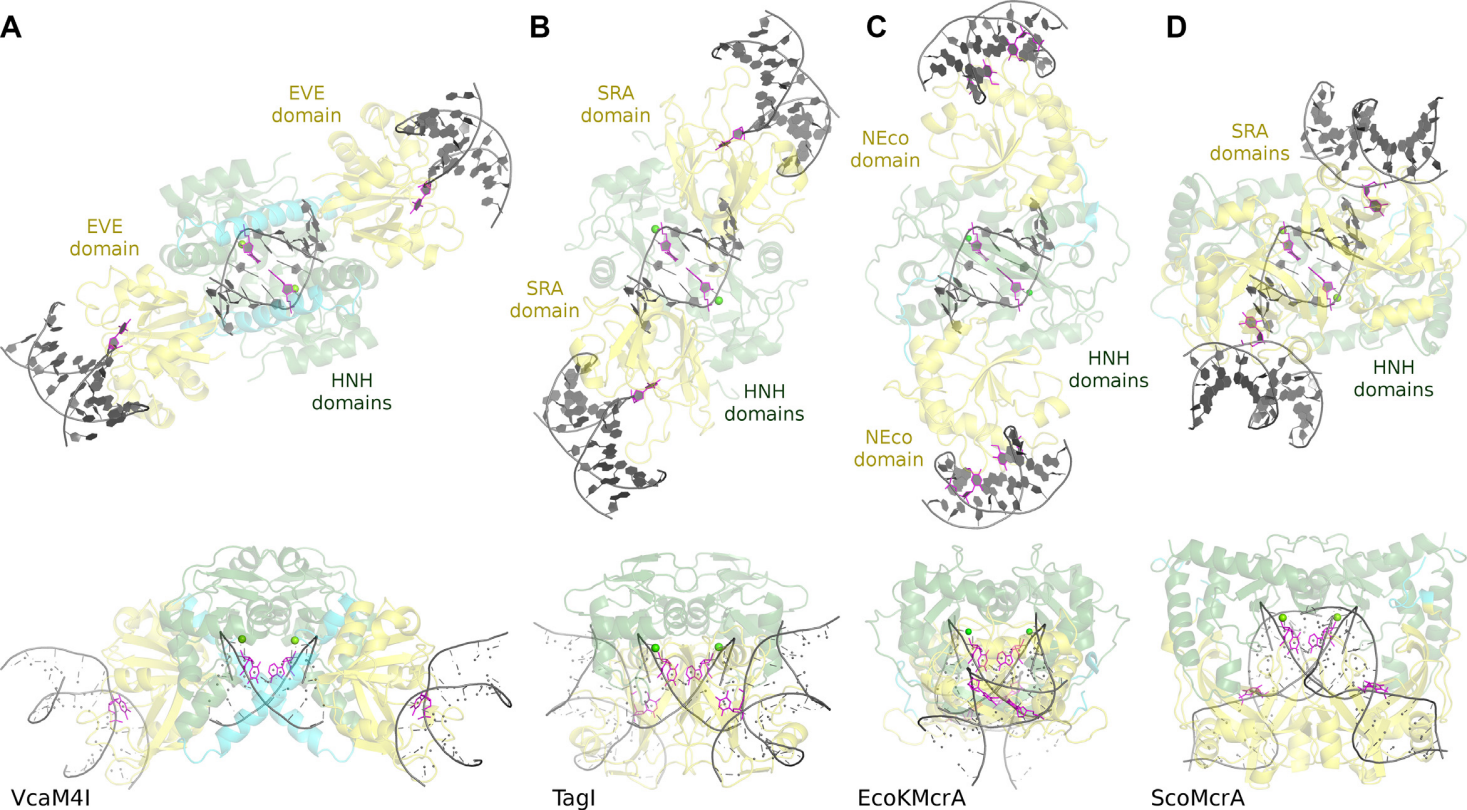
DNA binding by MDREs. DNA fragments bound to the catalytic HNH domains were modelled. The DNA binding modes to the PUA (exemplified by SRA and EVE) and NEco domains were determined experimentally except for TagI, which was modelled based on the UHRF1-DNA complex (PDB ID 3clz, (9)). For EcoKMcrA and ScoMcrA, the complexes of the isolated domains with bound DNA were mapped back onto the DNA free full-length enzyme structures. The EcoKMcrA structure was symmetrized based on the relative domain orientation in one of the protomers. In all cases there is no way to connect the DNA fragments bound to the catalytic and modification binding sites. There is also no easy way to extend the HNH domain bound DNA due to the clashes with the other domains. The repositioning of the non-catalytic parts of the enzymes would be necessary for the connection of the fragments and/or DNA extension.

While the DNA could be well accommodated in VcaM4I HNH domain binding groove, it could not be modelled in the structure of the full-length enzyme due to severe steric clashes with the inter-domain linker regions and the EVE domains (Figure 8A and 9A). These findings suggest that all crystal structures of VcaM4I in this work show catalytically inactive conformations, which are auto-inhibited by steric conflict. If this idea was correct, then a dimer of HNH domains (residues 179–309) alone should be highly active and toxic to cells, whereas the dimer of HNH domains with helical linker regions (residues 148–309), should be neither active nor toxic. To test this prediction, we attempted to transform *E. coli* (λDE3) cells with pET28a expression plasmid that was either empty or contained the HNH domain with linker region, the wild-type HNH domain, or as a control its inactive H224A variant (with His-SUMO tags). With the exception of the wild-type HNH domain, which was highly toxic to cells, the expression plasmids could be transformed with similar, high efficiency (Figure 8B). The expression construct for the HNH domain remained toxic even when T7 promoter leakage was decreased by including glucose in the media. Further experiments showed that the construct encoding the HNH domain with helical linker can be expressed with high yield. Gel filtration experiments confirmed that the HNH domains with linkers formed dimers, just as the control HNH domains alone (with H224A replacement, so that the domain could be expressed) (Supplementary Figure S18). We conclude from this experiment that the linker helix has auto-inhibitory role. We presume that in solution, this helix adopts a different conformation once long DNA with 5mC or 5hmC is bound, even though this is not observed with the short oligoduplexes present in the crystals.

### Small angle X-ray scattering (SAXS) experiments to probe VcaM4I conformation in solution

The auto-inhibition model suggests that VcaM4I may change conformation once dsDNA with a 5mC or 5hmC modification is bound to an EVE domain. To test this model, we carried out SAXS experiments for either VcaM4I alone or the enzyme in complex with the 11-, 32-, 35- and 39-mer blunt ended dsDNA molecules in the presence of EDTA that ligates divalent metal ions and thus prevents cleavage of the substrate (Supplementary Table 2). In all cases, one dsDNA molecule was present per VcaM4I dimer. The short 11-mer oligo contained a single 5mC base in the center (the same duplex as used for crystallization). Longer dsDNA molecules had two 5hmC bases in opposite strands near the ends of the DNA duplex, separated by 20, 23 and 27 base pairs, respectively. They were designed in the hope that VcaM4I may bind both modifications of one oligo simultaneously, and may thus be coaxed into adopting a DNA cleavage competent conformation.

The solution scattering data for the VcaM4I DNA mixtures fell into two broad classes. The VcaM4I alone and with 11-mer duplex generated scattering profiles that indicated ‘compact’ structures judging from the Kratky plot. This was not the case for the VcaM4I complexes with long dsDNAs and these duplexes alone. For VcaM4I–long dsDNAs complexes, the radii of gyration were in excess of 6 nm, even though the radii of VcaM4I and the dsDNAs were only 2.9 nm and between 2.9 and 3.5 nm, respectively. We conclude from these observations that VcaM4I in complex with the long dsDNAs forms aggregates, presumably because the DNA with two binding sites serves as a ‘cross-linker’ between the protein dimers. This aggregation effect makes it impossible to deduce conformational information from the scattering data.

To understand the data for VcaM4I–11-mer dsDNA complex, we used the program CRYSOL (28) to calculate theoretical scattering profiles for VcaM4I alone, VcaM4I mixed with but not bound to 11-mer dsDNA, VcaM4I with dsDNA bound to one EVE domain, a mixture of 50% of VcaM4I alone and 50% VcaM4I with dsDNA bound to both EVE domains, VcaM4I with modelled dsDNA bound to the catalytic domain and VcaM4I with dsDNA bound to both EVE domains. The theoretical curves show that it is possible to distinguish between dsDNA in solution, bound to EVE domains, or bound to the catalytic domains. However, the scattering curves for VcaM4I with dsDNA either present in one EVE domain per VcaM4I dimer, or a mixture of free VcaM4I and VcaM4I with two dsDNA molecules bound are too similar to be distinguished (Supplementary Figure S19).

Theoretical scattering curves calculated with CRYSOL (28) were compared with experimental scattering curves obtained using the program OLIGOMER (29). At low concentration, the scattering data for VcaM4I and its complex with dsDNA were well modelled by the crystallographic structures (for the enzyme alone, with dsDNA bound to one EVE domain, or the mixture model) (Supplementary Figure S19). However, we noticed that the χ^2^ values, which measure the degree of agreement between observed and predicted scattering data, deviated increasingly from the ideal value of 1 as the sample concentration got higher (reaching χ^2^ ∼7 for VcaM4I with dsDNA at 6.9 mg/ml concentration). Most likely, the imperfect fit is a consequence of slight, but systematic discrepancies in the high resolution region of the SAXS curve that receive more weight when the high resolution data are measured with better accuracy for more concentrated samples. Attempts to obtain a better fit at high resolution with alternative models, created based on normal mode analysis of the VcaM4I structures using the program elNEMO (41) were not successful.

## DISCUSSION

### EVE domains as base flipping sensors for 5mC or 5hmC

EVE domains are primarily known for their role in RNA biology (16). The crystal structures of VcaM4I in complex with single- and double-stranded DNA presented here indicate that the domains may also be used for binding of modified DNA. This finding validates the results of previous bioinformatics and biochemical screen suggesting that EVE domains can bind modified cytosine bases in DNA (19). VcaM4I detects the modification by flipping of the modified residue and accommodating it in the dedicated pocket, as most clearly demonstrated by the structure of its complex with dsDNA. The higher resolution crystal structures of VcaM4I with ssDNA containing 5mC or 5hmC show the details of the interaction with the flipped base with confidence. Single-stranded DNAs are bound very similarly to the proximal strand of dsDNA (Supplementary Figure S2).

A pocket in the EVE domain had been suggested in the original work defining this domain, based on amino acid conservation and the binding of ligands from crystallization buffers (16). The VcaM4I structure shows that this pocket was correctly identified, even though out of two previously emphasized residues of the EVE domain from *Pseudomonas syringae* (W26 and Y82; PDB ID: 2eve), one is not conserved and the other one is conservatively replaced by W82 in VcaM4I. The binding mode of the dsDNA to VcaM4I is also consistent with a prior prediction based on a co-crystal structure of THY28 with dsDNA (PDB ID: 5j3e) and a model of the protein with the flipped-out base (19). The modelling is however not precise enough to unequivocally pinpoint the functional residues (Figure 2 and Supplementary Figure S20).

### Comparison of the ligand binding modes of EVE and SRA domains

EVE and SRA domains belong to the PUA superfamily. The structures in this work together with representative prior structures of SRA domains with bound DNA (8–10) show that the DNA binding domains in the two families have more in common. They bind modified DNA on the same face and in similar orientation. They both flip modified 5mC or 5hmC bases and accommodate the flipped bases in pockets that are located in equivalent places in the common scaffold (Figure 2A, B). However, the nature of the pocket for the flipped base is quite different. Compared to the prototypical SRA domain pockets, the EVE domain pocket of VcaM4I is generally more hydrophobic, and also engages in more specific interactions with the Watson-Crick edge of the flipped base. Intercalating residues filling the stack of DNA bases, and interactions with the estranged base are also different (Figure 4).

Based on the hydrophobic nature of the pocket for the flipped base in the EVE domain of VcaM4I, and the greater similarity of its pocket to the one of UHRF1 compared to UHRF2 (Figure 4), one may expect a preference for 5mC over 5hmC containing DNA. In our original EMSA assays, we indeed observed a slight VcaM4I preference in this direction. However, this difference did not occur consistently, and in most experiments, we observed a comparable preference for either 5mC or 5hmC containing over non-modified DNA (Supplementary Figure S8). This result contrasts with 5hmC being clearly favored over 5mC in the cleavage assays performed for VcaM4I (Supplementary Figure S10) and other previously described EVE-HNH endonucleases (19). It is known that cytosine methylation changes biophysical properties of DNA. Upon cytosine methylation, it becomes not only more hydrophobic, but also changes structurally (42). It is further known that oxidation of 5mC to 5hmC reverses at least some methylation associated DNA property changes (43). We suspect that VcaM4I is recruited equally well to 5mC and 5hmC containing DNA by the EVE domain, but then digests the 5hmC containing DNA more efficiently with its nuclease domain, because of a preference of the nuclease domain for the less ‘atypical’ DNA. Unfortunately, we were not able to test this model directly, because the active version of the HNH domain in isolation could not be expressed in *E. coli* due to its toxicity.

SRA domains vary in sequence specificity. While some are believed to be only modification specific, others are known to have at least some context specificity (44). For the EVE domain of VcaM4I, there are no experimental data on the possible sequence specificity, and the crystal structures do not show obvious contacts that would predict it. We therefore expect that the VcaM4I EVE domain has either no or only very limited modification context dependence.

### Overall architecture of VcaM4I is typical for NTP-independent MDREs

The NTP-independent, modification dependent restriction endonucleases tend to share common two domain architecture, characterized by modification-sensing domains, and nuclease domains that in most, but not all cases, are believed to be themselves modification independent. Irrespective of whether the modification sensing domains are N-terminal, as in VcaM4I and most other two domain MDREs, or C-terminal, as in the PvuRts1I family (2), the proteins typically form dimers via contacts of the nuclease domains. This makes sense, because the dimeric nature of the nuclease domains helps with catalysis of double-strand breaks and the presence of two modification binding domains should increase the preference for modified over unmodified DNA. Among HNH nuclease domain dimers, the similarities go further. Dimerization interfaces, contributed in part of by the α-helices of the ββα-core motifs, are also similar (Supplementary Figure S3), and may explain the conserved stagger of the catalyzed DNA cleavages (resulting in products with single nucleotide 3′-overhangs).

Double-stranded DNA molecules that are bound to the modification sensing domains and the HNH domains of MDREs typically cannot be easily connected (Figure 9), given the ∼35 nm or ∼100 bp persistence length of dsDNA (45). VcaM4I is no exception to this rule: the observed conformations suggest either that the activation must happen in *trans*, or that major conformational changes must occur. For at least some SRA-HNH (TagI) (3) and PD-(D/E)XK-SRA (PvuRts1I) (46) restriction endonucleases, it has been shown that two appropriately spaced modified nucleobases stimulate cleavage more effectively than a single modified nucleobase. For VcaM4I, this has not yet been studied, and so it is unclear whether DNA cleavage (for low concentration) is promoted by the presence of two modified DNA bases and if so what their relative location should be.

## DATA AVAILABILITY

The final model coordinates and the corresponding structure factors were deposited in the Protein Data Bank with the following accession codes: 6YEX (VcaM4I in the absence of DNA), 6YJB (VcaM4I-5hmC_ssDNA complex) 6YKF (VcaM4I-5mC_ssDNA complex) and 6YMG (VcaM4I-dsDNA complex).

## Supplementary Material

gkaa1218_Supplemental_File
