# Supplementary material for: Crystal structures of the EVE-HNH endonuclease VcaM4I in the presence and absence of DNA
Source: Nucleic Acids Res. 2021 Jan 15;49(3):1708–23. doi: 10.1093/nar/gkaa1218 (PMC7897488; doi:10.1093/nar/gkaa1218)
Supplement: gkaa1218_Supplemental_File [file gkaa1218_supplemental_file.pdf]

Supplementary Materials for:

Crystal structures of the EVE HNH endonuclease VcaM4I in the presence and absence of DNA

Michal Pastor[1,2], Honorata Czapinska[1], Igor Helbrecht[1,2], Katarzyna Krakowska[1], Thomas Lutz[3], Shuang-yong Xu[3,#], Matthias Bochtler[1,2,#]

[1]*International Institute of Molecular and Cell Biology, Trojdena 4, 02-109 Warsaw, Poland*

[2]*Polish Academy of Sciences, Institute of Biochemistry and Biophysics, Pawinskiego 5a, 02-106 Warsaw, Poland*

[3]*New England Biolabs, Inc. 240 County Road, Ipswich, MA 01938, USA*

[#] Correspondence to: mbochtler@iimcb.gov.pl and xus@neb.com

Keywords: PUA superfamily, EVE domain, modification-dependent restriction endonuclease, 5-hydroxymethylcytosine, 5-methylcytosine, crystal structure

Supplementary Material contains: Figures, Tables, and Raw data.

Supplementary Figures

Suppl. Fig. S1. Purification of VcaM4I and its variants. (A) First batch of VcaM4I mutants. The purity of VcaM4I and its variants was assayed by PAGE. W22A and N241A could not be obtained, likely due to the folding issues or premature termination. W82A migrated on the gel slightly slower than the wild-type (WT) enzyme. The reason for this is unknown. Y130A has lower protein yield and higher background due to its toxicity to the expression host. The molecular mass of the monomeric VcaM4I is predicted to be 35.6 kDa. M - protein ladder (NEB). **(B)** Second batch of VcaM4I mutants with more drastic amino acid (aa) substitutions. Y130W has lower protein yield and higher background due to its toxicity to the expression host.

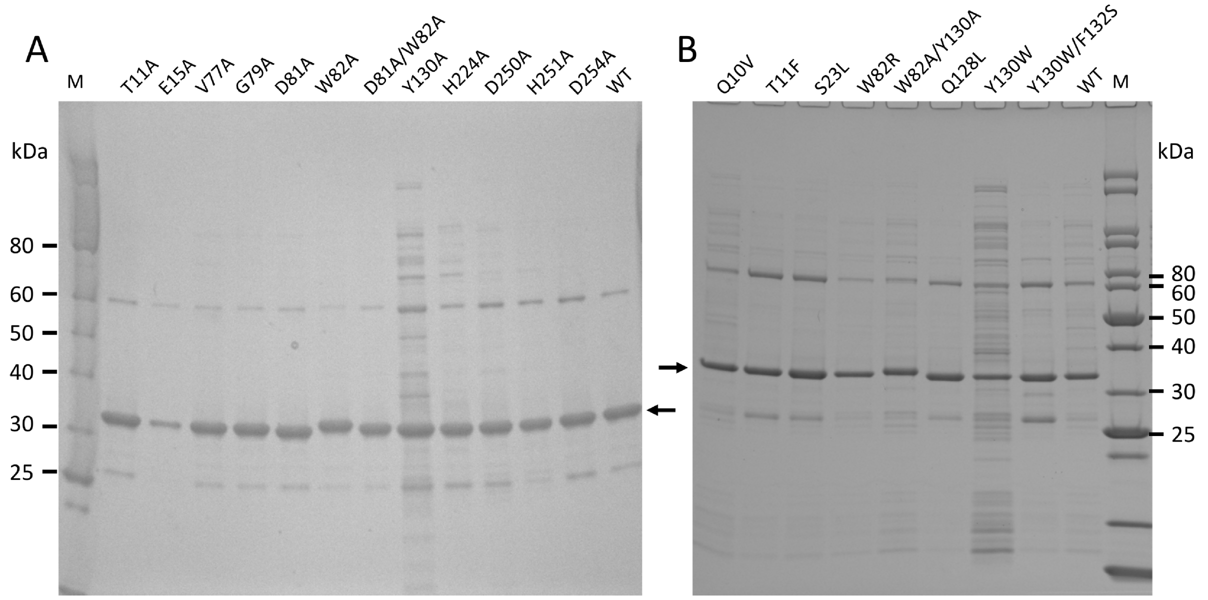

Suppl. Fig. S2. Comparison of the VcaM4I and its DNA complexes. (A) VcaM4I in the presence (colored) and absence (white) of the dsDNA, **(B)** the conformation of double- (colored) and single- (gray) stranded DNA in complex with the enzyme, and **(C)** comparison of the binding mode of methylated (dark) and hydroxymethylated (light) ssDNA to VcaM4I.

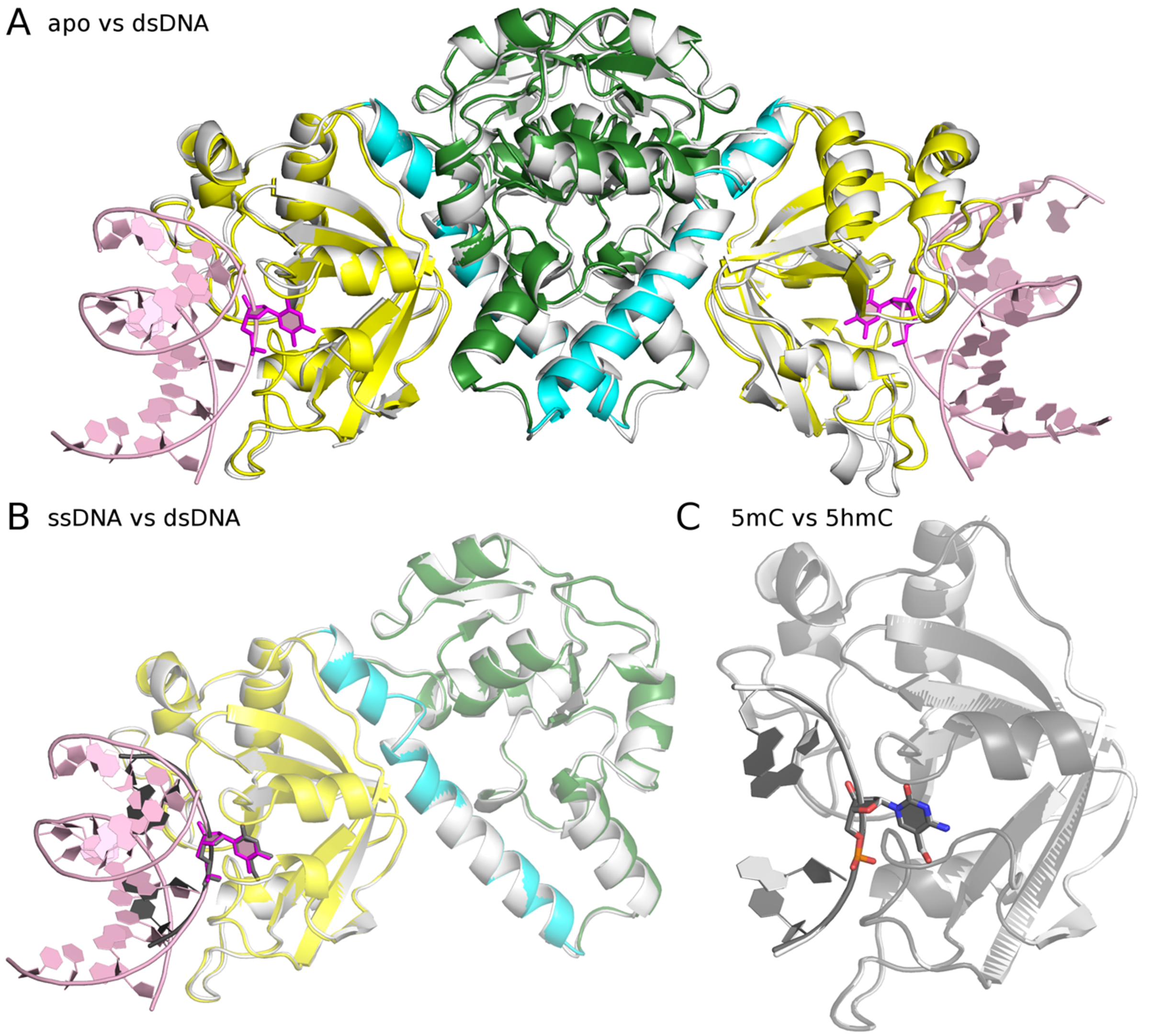

Suppl. Fig. S3. Conserved dimerization interface of HNH restriction endonucleases. The DNA complex models of catalytic domain dimers of four restriction enzymes: **(A)** VcaM4I, **(B)** TagI (PDB 6ghs (1)), **(C)** EcoKMcrA (PDB 6ghc (2)) and **(D)** ScoMcrA (PDB 5zmm (3)) were generated based on the Hpy99I-DNA structure (4). Each DNA strand was first separately mapped based on the active site overlay. The DNA from Hpy99I complex (severely bent due to the insertion of the helices of the ββα core motif into the DNA minor groove) was then overlaid on the isolated strands to regenerate the base pairing. The orientation of the DNA was manually optimized to eliminate clashes with the protein and to improve the location of the active sites with respect to the scissile phosphoester bonds. The obtained models were in almost perfect agreement with the model based on low resolution colicin E9 DNA structure described in the manuscript. The metal ions were modelled in the VcaM4I and ScoMcrA structures in which they were missing. In agreement with the experimental information all presented enzymes are predicted to generate 1 nucleotide 3′-overhangs.

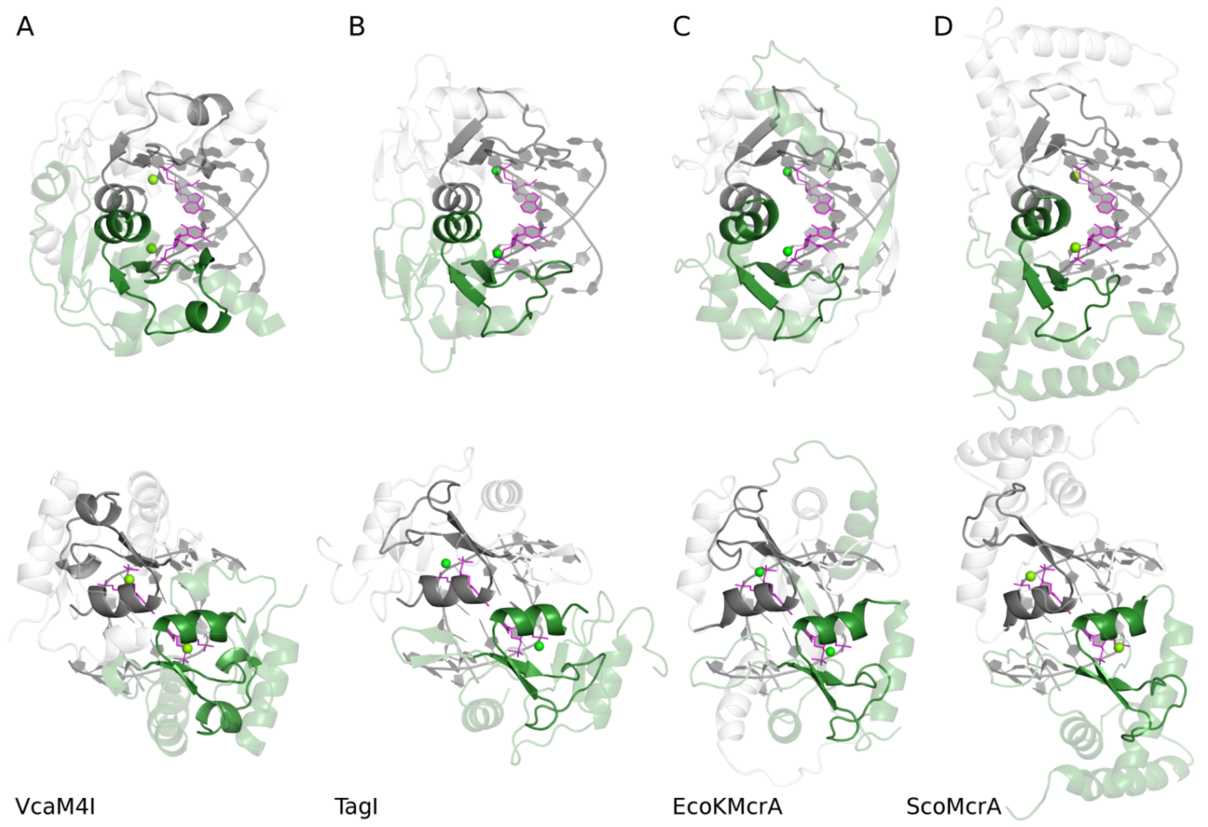

Suppl. Fig. S4. Topology diagrams of selected PUA-superfamily domains. The typical domain core that gives rise to family classification (EVE, YTH, ASCH, and PUA) is presented in color on the left. The PDB IDs of the structures from which the domains are derived are indicated below the diagrams.

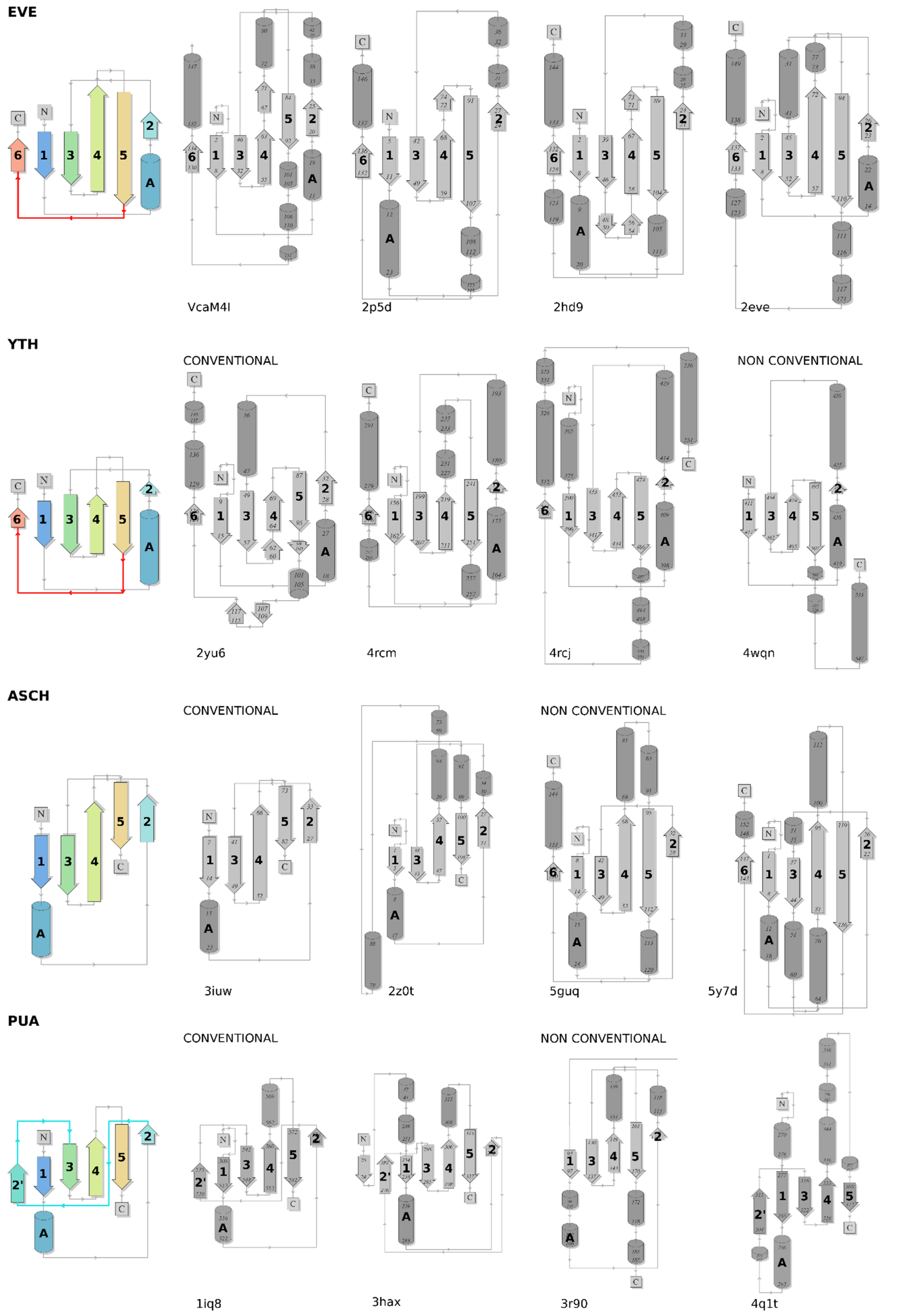

Suppl. Fig. S5. Structure based sequence alignment generated with the DALI server (5). The MSA was the basis for Suppl. Table S2. PDB code, Z-score, RMSD and alignment length are indicated before the protein names. The columns in the alignment corresponding to the gaps in the VcaM4I sequence were removed. Z-score cutoff of 4.0 was applied and the structures with more than 50% sequence identity were eliminated.

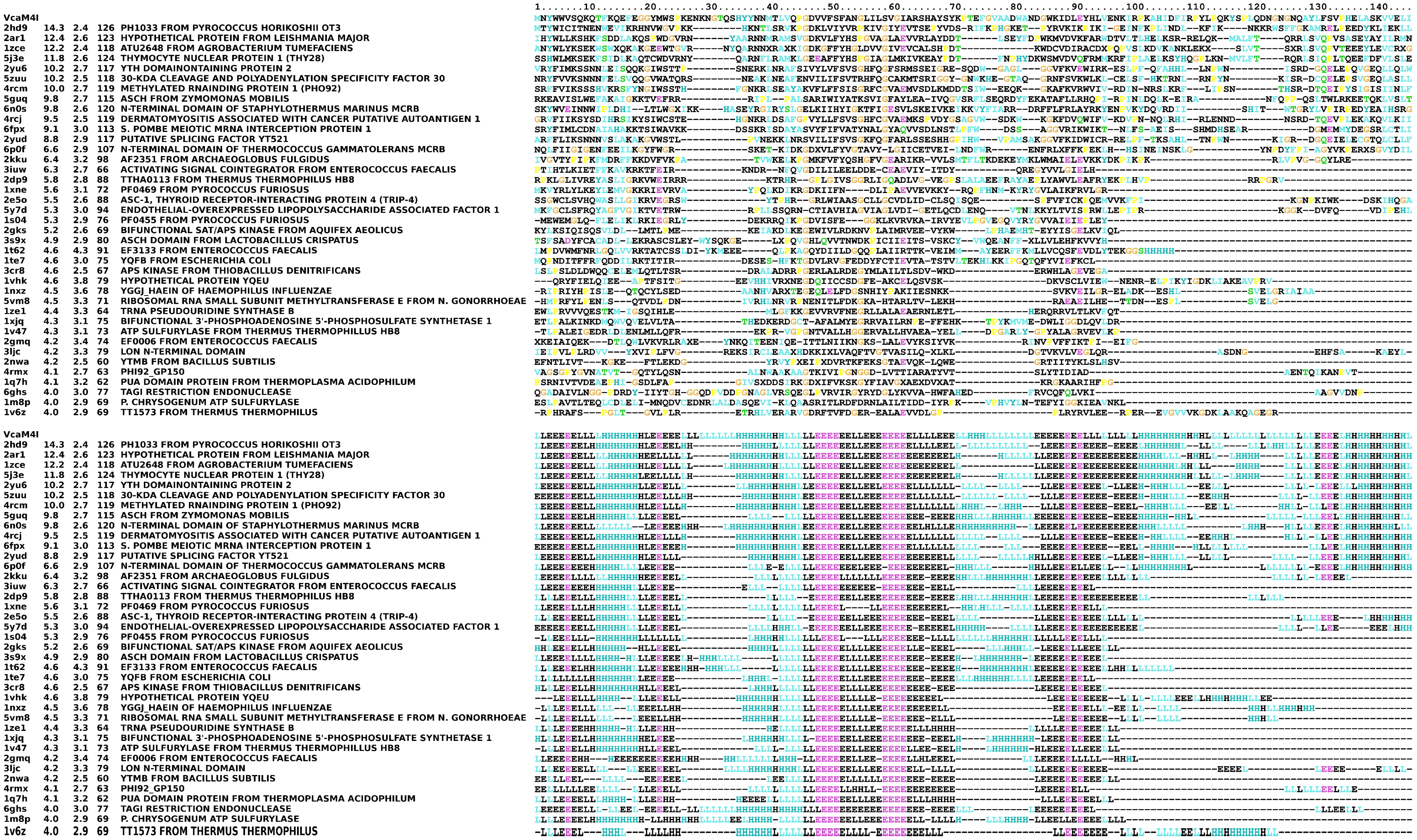

Suppl. Fig. S6. Taxonomic distribution of the EVE domain containing proteins present in the sequence alignment underlying the conservation score mapping presented in Fig. 3B. More than 90% of sequences were annotated as HNH endonucleases. The alignment was generated automatically with the default ConSurf server settings (multiple sequence alignment built using MAFFT, homologues collected from UNIREF90, HMMER homolog search algorithm, HMMER E-value: 0.0001, 1 HMMER iteration, max. % ID between sequences: 95, minimal % ID for homologs: 35, 150 sequences that sample the list of homologues to the query, Bayesian conservation scores calculation method, best fit model of substitution for proteins).

- root (130 nodes)
  - bacteria (127 nodes)
    - proteobacteria (112 nodes)
      - γ-proteobacteria (52 nodes)
        - enterobacteria (1 node)
        - other γ-proteobacteria (51 nodes)
      - α-proteobacteria (29 nodes)
      - β-proteobacteria (28 nodes)
      - δ-proteobacteria (3 nodes)
    - CFB group bacteria (3 nodes)
    - verrucomicrobia (2 nodes)
    - cyanobacteria (2 nodes)
    - high GC Gram+ (1 node)
    - firmicutes (1 node)
    - other bacteria (6 nodes)
  - euryarchaeotes (1 node)
  - other (2 nodes)

Suppl. Fig. S7. Sequence conservation mapping and taxonomic distribution of the proteins present in the multiple sequence alignment generated with significantly relaxed parameters. The VcaM4I surface was colored from lowest to highest conservation scores in turquoise to violet red color scale as in Fig. 3B. The parameters altered with respect to the ones stated in **Suppl. Fig. S6** are indicated above the figures. The flipped base binding pocket and often also the intercalating residue are conserved even in alignments generated with very generous criteria for inclusion of distantly related amino acid sequences.

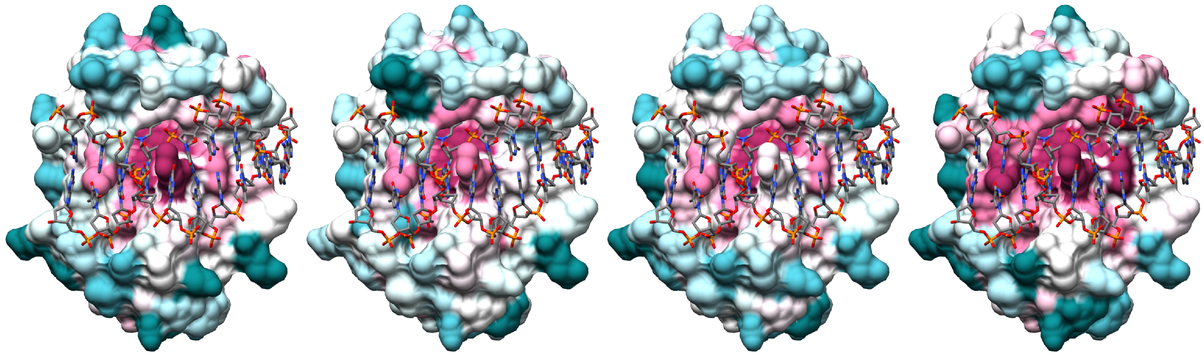

seq500 maxid40 minid15 e0.01 runs5

- root (423 nodes)
  - bacteria (341 nodes)
    - proteobacteria (155 nodes)
      - α-proteobacteria (75 nodes)
      - γ-proteobacteria (45 nodes)
        - enterobacteria (6 nodes)
        - other γ-proteobacteria (39 nodes)
      - β-proteobacteria (20 nodes)
      - δ-proteobacteria (11 nodes)
      - other proteobacteria (3 nodes)
    - firmicutes (64 nodes)
    - actinobacteria (36 nodes)
      - high GC Gram+ (35 nodes)
      - other actinobacteria (1 node)
    - CFB group bacteria (19 nodes)
    - GNS bacteria (10 nodes)
    - cyanobacteria (10 nodes)
    - verrucomicrobia (8 nodes)
    - spirochetes (1 node)
    - planctomycetes (1 node)
    - other bacteria (36 nodes)
  - eukaryotes (49 nodes)
    - fungi (24 nodes)
      - ascomycetes (15 nodes)
      - basidiomycetes (9 nodes)
    - animals (12 nodes)
      - chordates (8 nodes)
        - mammals (5 nodes)
          - rodents (2 nodes)
          - bats (1 node)
          - carnivores (1 node)
          - other mammals (1 node)
        - frogs & toads (1 node)
        - lizards (1 node)
        - other chordates (1 node)
      - nematodes (2 nodes)
      - arachnids (1 node)
      - gastropods (1 node)
    - flowering plants (12 nodes)
    - diatoms (1 node)
  - archaea (27 nodes)
    - euryarchaeotes (22 nodes)
    - crenarchaeotes (1 node)
    - other archaea (4 nodes)
  - other (5 nodes)

seq500 maxid50 minid15 e0.01 runs5

- root (438 nodes)
  - bacteria (365 nodes)
    - proteobacteria (113 nodes)
      - γ-proteobacteria (50 nodes)
        - enterobacteria (7 nodes)
          - enterobacteria (1 node)
          - other enterobacteria (6 nodes)
        - other γ-proteobacteria (43 nodes)
      - α-proteobacteria (34 nodes)
      - β-proteobacteria (13 nodes)
      - δ-proteobacteria (9 nodes)
      - ε-proteobacteria (1 node)
      - other proteobacteria (5 nodes)
    - firmicutes (86 nodes)
    - actinobacteria (79 nodes)
      - high GC Gram+ (77 nodes)
      - other actinobacteria (2 nodes)
    - CFB group bacteria (30 nodes)
    - verrucomicrobia (8 nodes)
    - GNS bacteria (7 nodes)
    - cyanobacteria (4 nodes)
    - spirochetes (3 nodes)
    - planctomycetes (1 node)
    - aquificales (1 node)
    - fusobacteria (1 node)
    - green sulfur bacteria (1 node)
    - other bacteria (30 nodes)
  - archaea (38 nodes)
    - euryarchaeotes (30 nodes)
    - crenarchaeotes (4 nodes)
    - other archaea (4 nodes)
  - eukaryotes (29 nodes)
    - fungi (16 nodes)
      - ascomycetes (10 nodes)
        - budding yeasts (2 nodes)
        - other ascomycetes (8 nodes)
      - basidiomycetes (6 nodes)
        - rust fungi (1 node)
        - smut fungi (1 node)
        - other basidiomycetes (4 nodes)
    - green plants (4 nodes)
      - flowering plants (2 nodes)
      - green algae (2 nodes)
    - animals (3 nodes)
      - nematodes (2 nodes)
      - rodents (1 node)
    - haptophytes (1 node)
    - kinetoplastids (1 node)
    - red algae (1 node)
    - ciliates (1 node)
    - diatoms (1 node)
    - other eukaryotes (1 node)
  - other (5 nodes)
  - other viruses (0 node)

seq500 maxid60 minid20 e0.01 runs5

- root (422 nodes)
  - bacteria (353 nodes)
    - proteobacteria (144 nodes)
      - γ-proteobacteria (58 nodes)
        - enterobacteria (6 nodes)
        - other γ-proteobacteria (52 nodes)
      - α-proteobacteria (42 nodes)
      - β-proteobacteria (20 nodes)
      - δ-proteobacteria (10 nodes)
      - other proteobacteria (13 nodes)
    - firmicutes (65 nodes)
    - actinobacteria (41 nodes)
      - high GC Gram+ (40 nodes)
      - other actinobacteria (1 node)
    - CFB group bacteria (29 nodes)
    - verrucomicrobia (11 nodes)
    - cyanobacteria (9 nodes)
    - GNS bacteria (7 nodes)
    - green sulfur bacteria (2 nodes)
    - planctomycetes (1 node)
    - aquificales (1 node)
    - other bacteria (41 nodes)
  - eukaryotes (41 nodes)
    - fungi (24 nodes)
      - ascomycetes (12 nodes)
        - budding yeasts (2 nodes)
        - other ascomycetes (10 nodes)
      - basidiomycetes (10 nodes)
        - rust fungi (1 node)
        - other basidiomycetes (9 nodes)
      - chytrids (1 node)
      - other fungi (1 node)
    - green plants (5 nodes)
      - flowering plants (4 nodes)
      - other green plants (1 node)
    - animals (4 nodes)
      - nematodes (2 nodes)
      - fleas (1 node)
      - even-toed ungulates (1 node)
    - red algae (3 nodes)
    - kinetoplastids (2 nodes)
    - haptophytes (1 node)
    - cryptomonads (1 node)
    - other eukaryotes (1 node)
  - archaea (24 nodes)
    - euryarchaeotes (21 nodes)
    - other archaea (3 nodes)
  - other (3 nodes)

seq500 maxid70 minid25 e0.01 runs5

- root (395 nodes)
  - bacteria (337 nodes)
    - proteobacteria (166 nodes)
      - γ-proteobacteria (66 nodes)
        - enterobacteria (1 node)
        - other γ-proteobacteria (65 nodes)
      - α-proteobacteria (46 nodes)
      - β-proteobacteria (23 nodes)
      - δ-proteobacteria (18 nodes)
      - ε-proteobacteria (1 node)
      - other proteobacteria (12 nodes)
    - firmicutes (37 nodes)
    - cyanobacteria (19 nodes)
    - CFB group bacteria (17 nodes)
    - high GC Gram+ (15 nodes)
    - verrucomicrobia (9 nodes)
    - GNS bacteria (7 nodes)
    - planctomycetes (2 nodes)
    - green sulfur bacteria (1 node)
    - other bacteria (61 nodes)
  - eukaryotes (42 nodes)
    - fungi (23 nodes)
      - ascomycetes (14 nodes)
        - budding yeasts (3 nodes)
        - other ascomycetes (11 nodes)
      - basidiomycetes (5 nodes)
      - chytrids (2 nodes)
        - monoblepharidomycetes (1 node)
        - other chytrids (1 node)
      - other fungi (2 nodes)
    - animals (8 nodes)
      - arthropods (3 nodes)
        - flies (1 node)
        - other arthropods (2 nodes)
      - mammals (2 nodes)
        - carnivores (1 node)
        - primates (1 node)
      - nematodes (2 nodes)
      - sponges (1 node)
    - green plants (6 nodes)
      - flowering plants (3 nodes)
      - green algae (2 nodes)
      - other green plants (1 node)
    - kinetoplastids (1 node)
    - cryptomonads (1 node)
    - diatoms (1 node)
    - other eukaryotes (2 nodes)
  - archaea (11 nodes)
    - euryarchaeotes (8 nodes)
    - other archaea (3 nodes)
  - other (3 nodes)

Suppl. Fig. S8. Electrophoretic mobility shift assay (EMSA) of wild-type VcaM4I in two repeats. 25 nM of dsDNA was used. The protein was incubated with the oligoduplexes for one hour before running the assay.

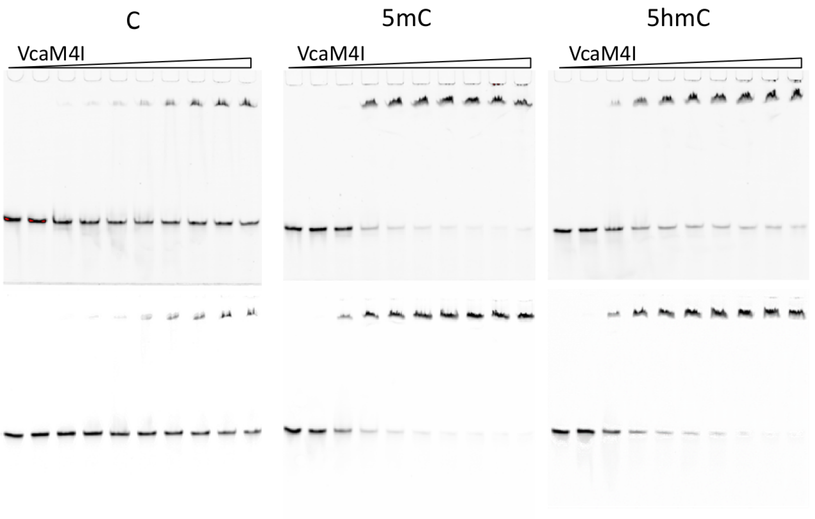

VcaM4I [nM]	C [% bound]	5mC [% bound]	5hmC [% bound]
1000	47	93	89
800	42	96	84
600	36	94	84
500	27	92	80
400	14	90	75
300	8	85	69
200	4	71	56
100	2	4	16
50	0	3	1

VcaM4I [nM]	C [% bound]	5mC [% bound]	5hmC [% bound]
1000	37	98	94
800	38	97	95
600	28	96	92
500	23	96	87
400	12	92	83
300	4	86	77
200	3	74	65
100	1	31	28
50	0	0	0

Suppl. Fig. S9. Electrophoretic mobility shift assay (EMSA) of VcaM4I variants. 25 nM of dsDNA was used. The protein concentrations were analogous as in Suppl. Fig. S8. The protein was incubated with the oligoduplexes for one hour before running the assay.

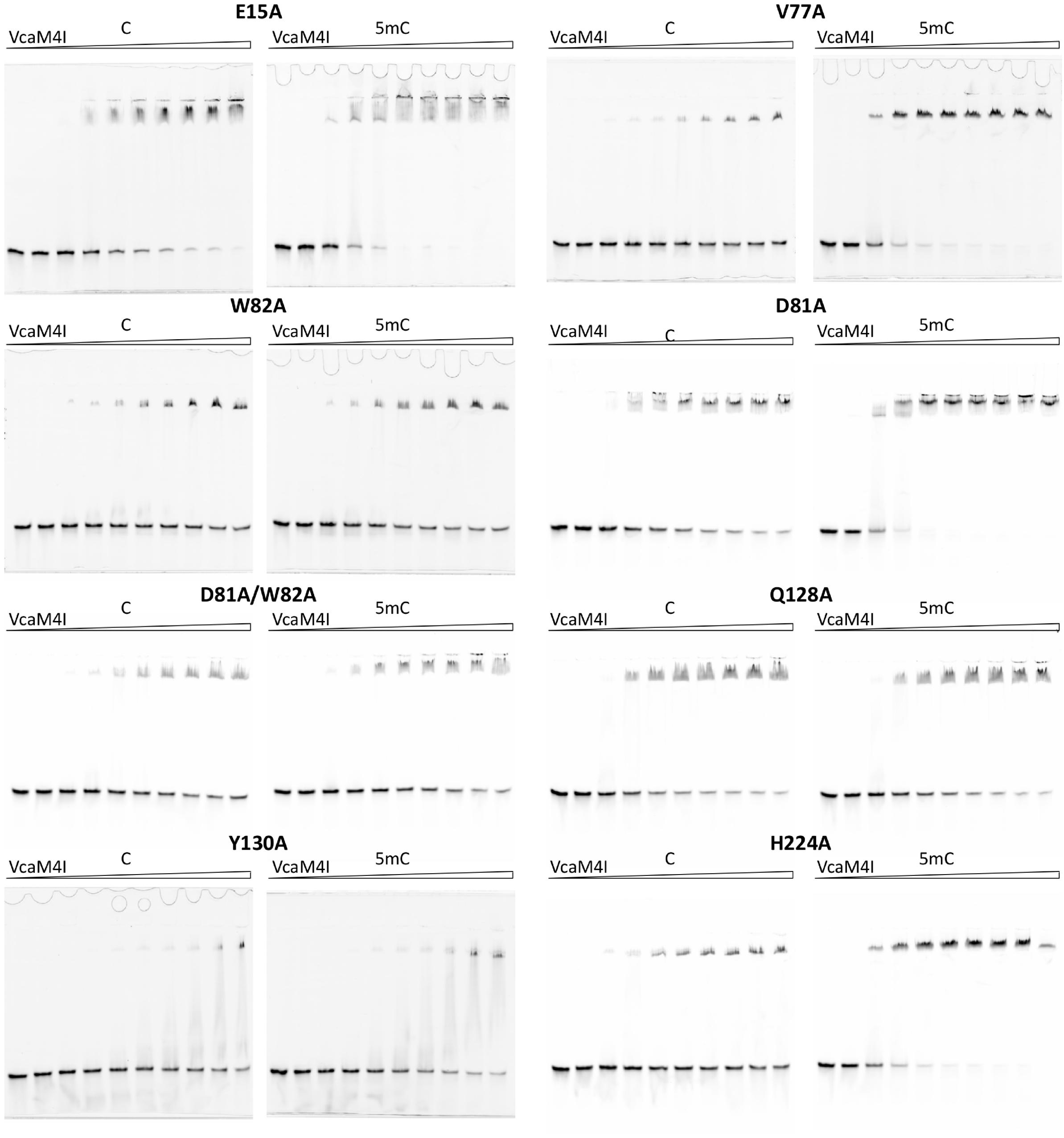

Suppl. Fig. S10. Modification dependence of VcaM4I activity in Mg^{2+} and Mn^{2+} containing buffers. The sizes of differentially modified dsDNA substrates were swapped between A and B to eliminate the possibility of DNA length effects. **(A)** The assay was performed in the presence of either Mg^{2+} (10 mM) and Mn^{2+} (1 mM) buffer with 50 mM NaCl. The concentrated wild-type enzyme stock was set at 1 mg/ml, which was diluted further (2 µl of 2-fold to 800-fold dilutions used in the digestions) to digest 8.1 nM DNA at 37 °C for 1 h. The protein concentrations were estimated assuming the active enzyme dimer of 71.2 kDa molar weight. **(B)** Fixed amount of PCR DNAs (8.1 nM) and decreasing concentrations of the enzyme were used in Mg^{2+} buffer. (-) marks the absence of the enzyme (uncut). The absence of bands or presence of fainter bands signalizes the nuclease activity.

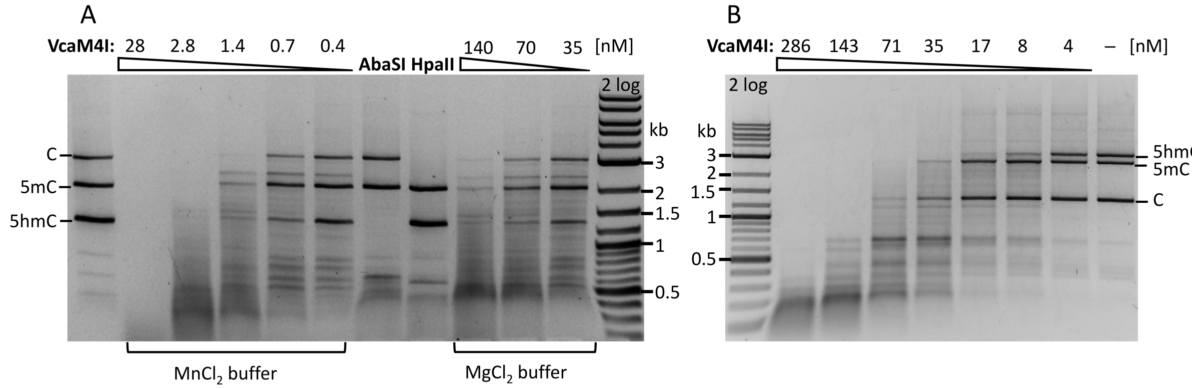

Suppl. Fig. S11. ***In vitro*** **endonuclease activity of primary EVE domain variants containing alanine substitutions on PCR DNA substrates**. **(A)** Activity of the primary set of VcaM4I variants in Mg^{2+}. The enzyme in decreasing concentration (~70, 35, 17 nM) was used to digest the PCR fragments (C - 1.1 kb, 5mC - 2.1 kb, 5hmC - 2.9 kb; 8.1 nM DNA) in NEB Buffer 2.1 (10 mM Mg^{2+}) for 30 min. **(B)** The substrate sizes were inverted when compared to (A) to confirm that the fragment size did not affect modification dependence (C - 3 kb, 5mC - 2.1 kb, 5hmC - 1.1 kb; 8.1 nM DNA). The low activity of E15A resulted in part from the overestimation of its concentration that was corrected for in (A). After the restriction reaction and before agarose gel analysis proteinase K was added to eliminate proteins. Residual short genomic DNA and RNA were carried-over from enzyme preparation. Known REases were used as controls: HpaII (cleaving unmodified DNA), PvuRts1I (cleaving 5hmC-containing DNA). (-) indicates uncut control. The absence of bands or presence of fainter bands signalizes the nuclease activity. The raw gels used to generate the figure are presented in Suppl. Raw Data below. WT VcaM4I appeared to cleave 5hmC DNA preferentially in Mg^{2+} buffer.

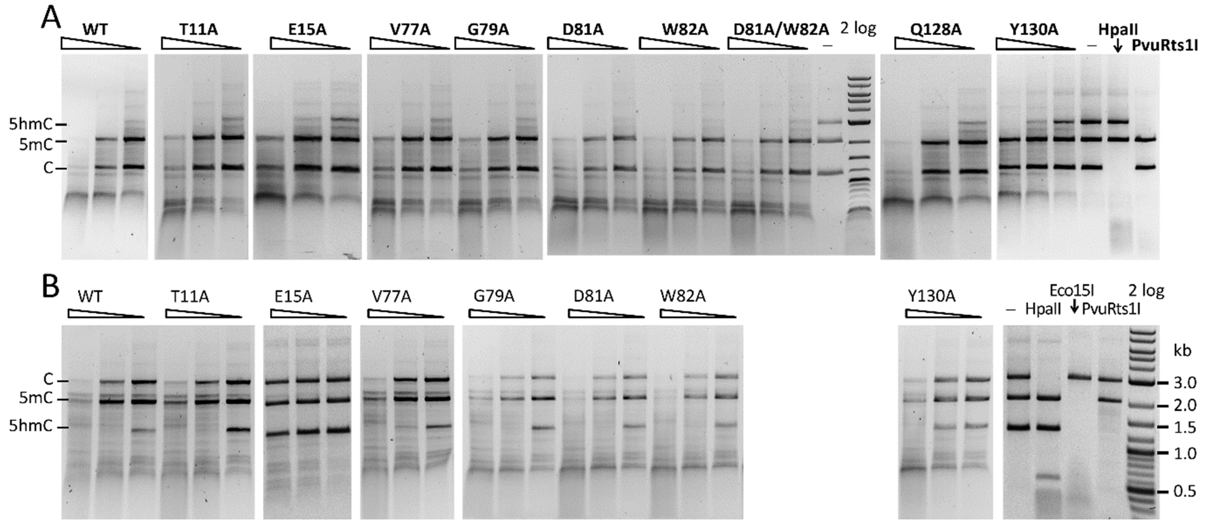

Suppl. Fig. S12. ***In vivo*** **restriction activity of VcaM4I and its primary variants in** **uninduced** **C2566** ***E. coli*** **cells**. The λvir (from Dam^+ Dcm^- strain - non-modified C), T4gt (5hmC) and T4 (g5hmC) phages were spotted onto the cell lawns of T7 Express cells expressing (without IPTG induction) pTXB1 plasmid that was empty, contained *tagIR* gene or contained *vcaM4IR* gene in the wild-type or mutated form. The 10^{-3}, 10^{-4} and 10^{-5} dilution series of were used for phage λvir, and 10^{-4}, 10^{-5} and 10^{-6} for T4gt and T4 phages. TagI endonuclease that restricts T4gt was used as a positive control. * The W22A variant used in this experiment had an additional frame-shift mutation.

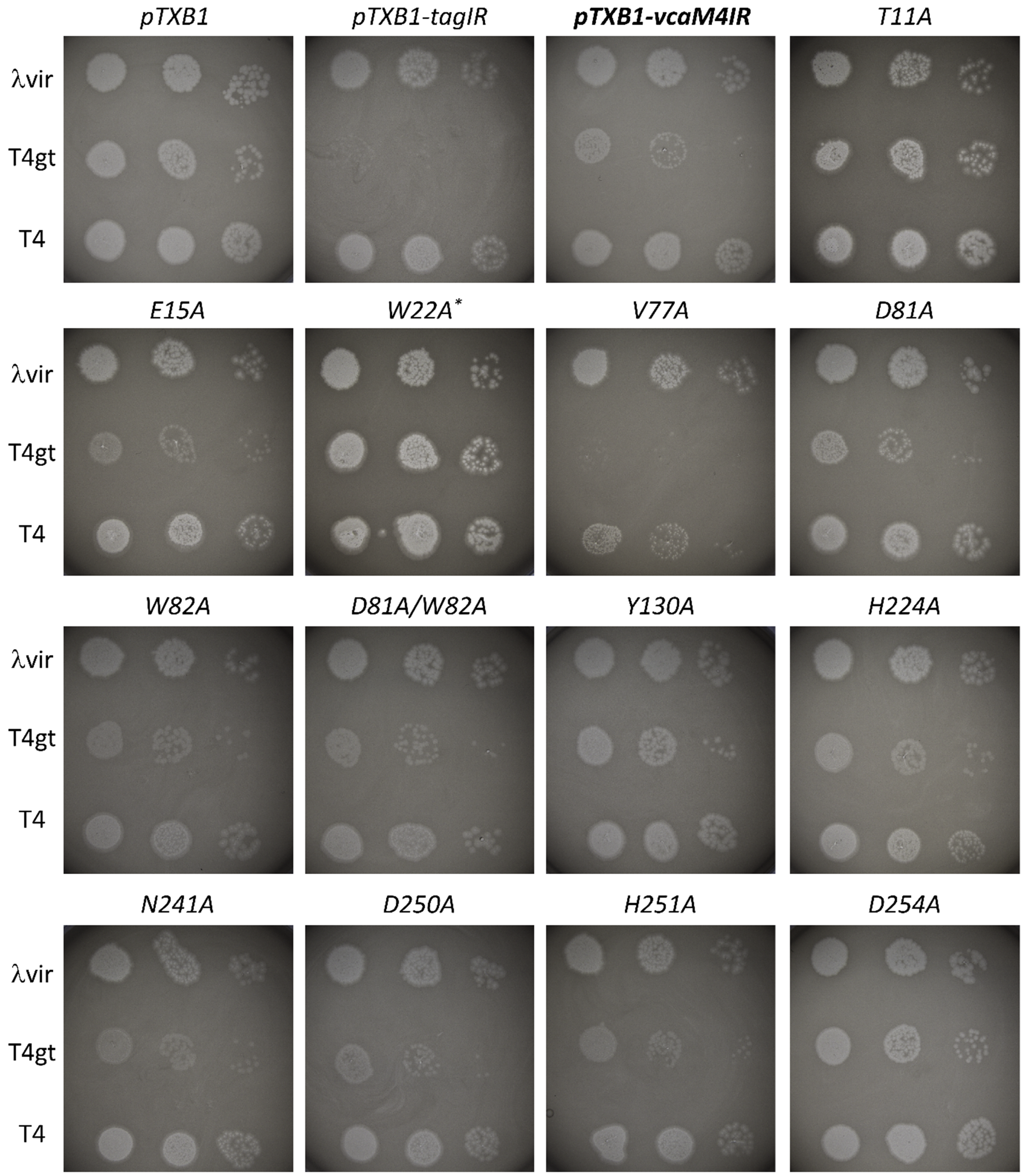

Suppl. Fig. S13. Restriction assay of the Q128A VcaM4I variant expressed in uninduced C2566 *E. coli* cells (complementary to Suppl. Fig. S12).

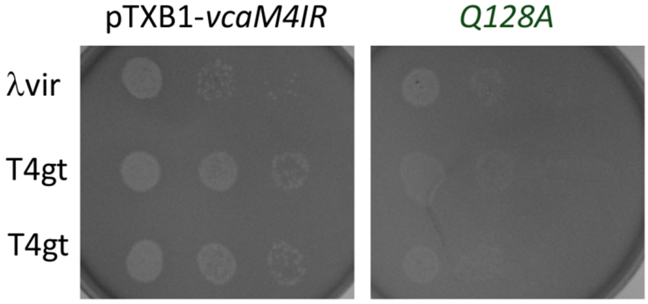

Suppl. Fig. S14. T4gt and λvir phage restriction in *E. coli* cells with IPTG-induced expression of VcaM4I and its primary variants. λvir phage dilutions equaled: 10^{-4}, 10^{-5}, 10^{-6}, T4gt dilutions: 10^{-5}, 10^{-6}, 10^{-7}. T4gt spot test was performed in duplicate to verify reproducibility.

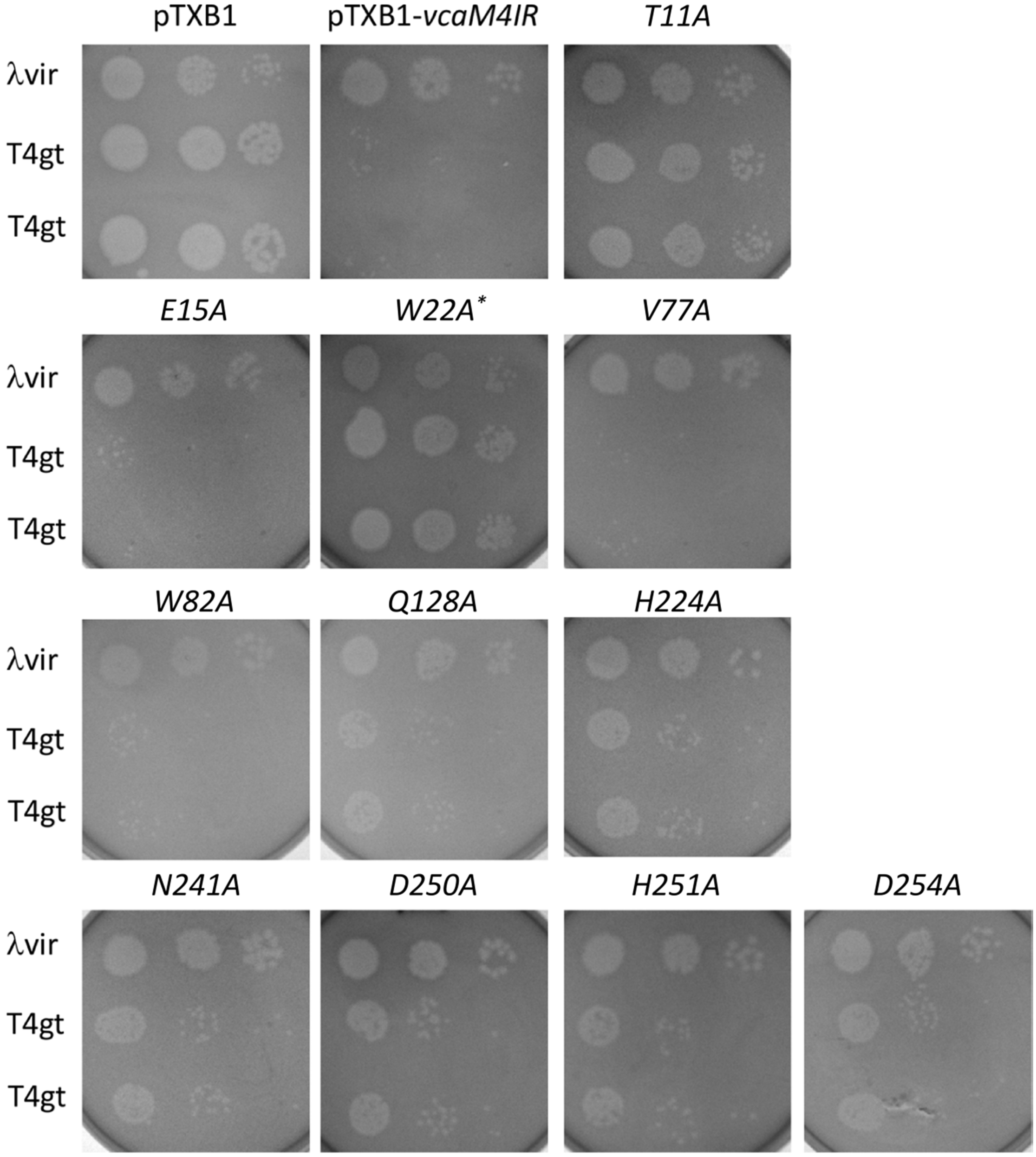

Suppl. Fig. S15. *In vitro* endonuclease activity of secondary EVE domain variants containing more aggressive (size increasing or charge altering) substitutions on PCR DNA substrates. **(A)** Activity of the secondary set of VcaM4I variants in Mg^{2+} buffer. Concentrated enzyme stock was set at 1 mg/ml. 2 µl of the enzyme in serial dilutions of 1/2 x (1 µg, ~280 nM), 1/4 (0.5 µg, ~140 nM) , 1/8 (0.25 µg, ~70 nM) in buffer containing (50 mM NaCl, 10 mM $MgCl_2$, 10 mM Tris-HCl, 1 mM DTT) digested 0.45 µg DNA mixture for 1 h at 37 °C. **(B)** Activity of the secondary set of VcaM4I variants in Mn^{2+} buffer. 2 µl of the enzyme in serial dilutions of 1 x (2 µg, ~560 nM), 1/10 (0.2 µg, 56 nM), 1/100 (0.02 µg, 5.6 nM), 1/200 (0.01 µg, ~2.8 nM) in buffer containing (50 mM NaCl, 10 mM Tris-HCl, 1 mM $MnCl_2$, 1 mM DTT) digested 0.45 µg DNA mixture for 1 h at 37 °C. Proteinase K was added after the restriction reaction to eliminate proteins. Residual short genomic DNA and RNA were carried-over from enzyme preparation. Known REases were used as controls: HpaII (cleaving unmodified DNA), AbaSI (cleaving 5hmC containing DNA) and MspJI (cleaving 5mC/5hmC DNA). (-) indicates control without added enzyme. The absence of bands or presence of fainter bands signalizes the nuclease activity. The raw gels used to generate the figure are presented in Suppl. Raw Data below.

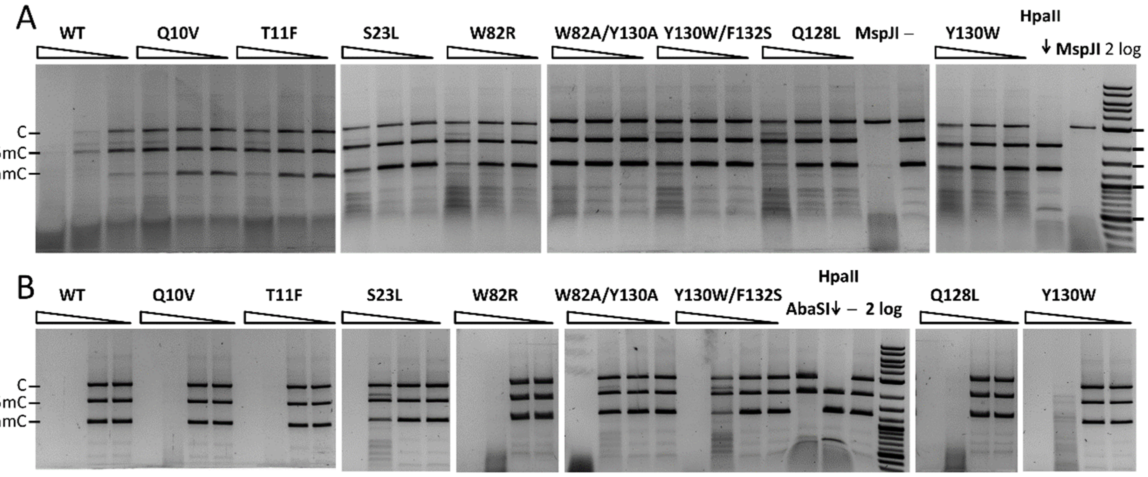

Suppl Fig. S16. *In vitro* activity of purified VcaM4I variants featuring changes in the HNH domain. PCR DNA fragment mixture (C – 2.9 kb, 5mC - 2.1 kb, 5hmC – 1.1 kb; 8.1 nM) was digested by the mutant enzyme (~70 and 35 nM) as indicated on top of each lane in NEB Buffer 2.1 (10 mM Mg^{2+}) for 30 min. Proteinase K was added after the reaction to remove proteins. Three restriction enzymes were used as controls: HpaII (cleaving unmodified DNA), Eco15I (cleaving 5hmC/5mC DNA) and PvuRts1I (cleaving 5hmC DNA). (-) indicates control without added enzyme. The absence of bands or presence of fainter bands signalizes the nuclease activity. The raw gel used to generate the figure is presented in Suppl. Raw Data.

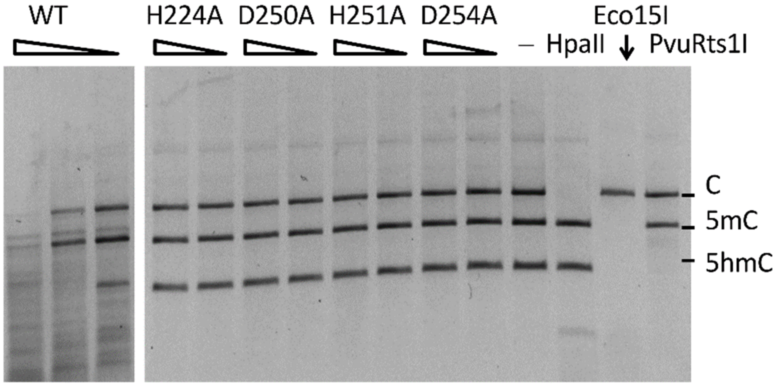

Suppl. Fig. S17. Predicted DNA binding mode of the VcaM4I HNH domain dimer and homologous fragments of other endonucleases. The electrostatic potential of the isolated HNH domain dimers of VcaM4I, TagI (PDB 6ghs (1)), EcoKMcrA (PDB 6ghc (2)) and ScoMcrA (PDB 5zmm (3)) was calculated with the help of the ABPS server (6). The predictions assumed the presence of divalent metal ions in the active sites and negatively charged binding sites for the structural Zn^{2+} ions. The DNA models were obtained as described for Suppl. Fig. S3. The active site metal ions absent from the VcaM4I and ScoMcrA structures were manually positioned in the vicinity of the catalytic residues. The fragments corresponding to residues 194-309 of VcaM4I (eliminating the first short helix of the domain that might unwind together with the linker helix). The charge distribution was mapped on the domain surfaces with CHIMERA program (7). They imply cleavage with one nucleotide 3′-overhangs in agreement with the experimental data and conserved dimerization mode (8). Please note that the figure is 180° rotated with respect to Fig. 7.

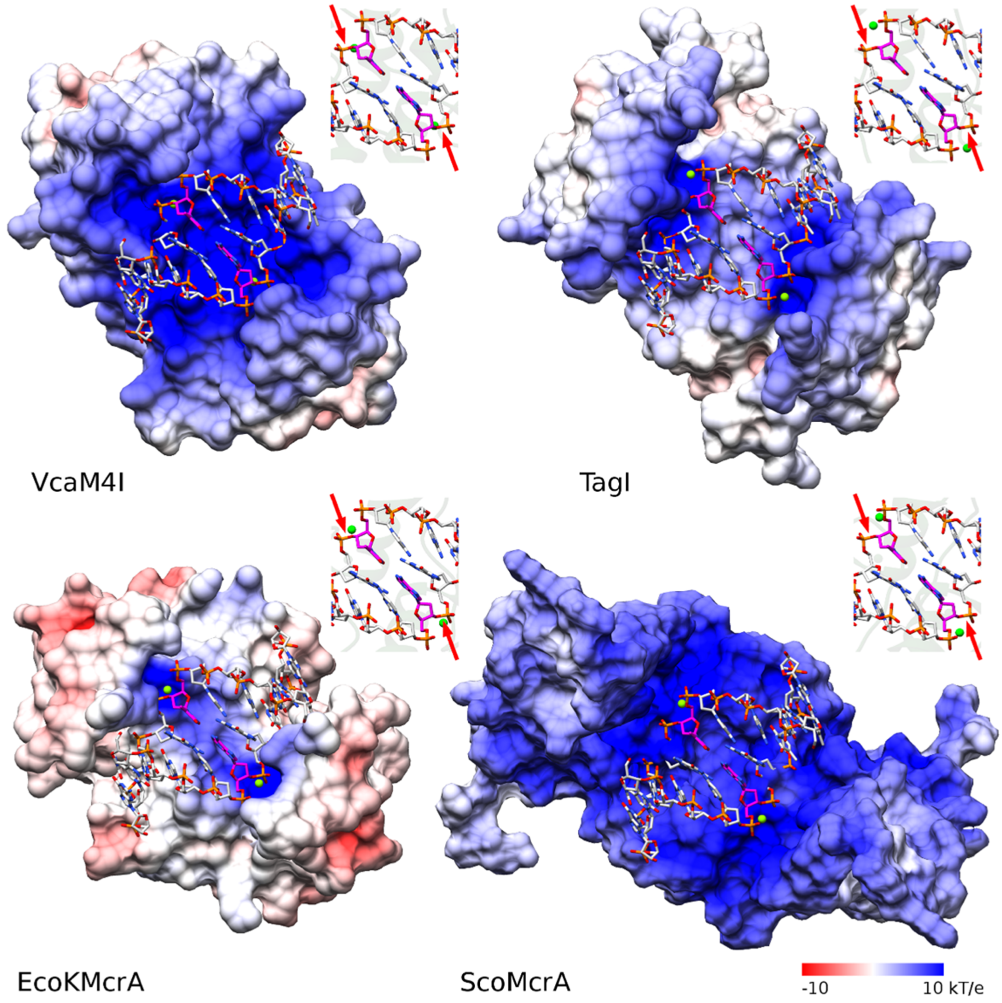

Suppl. Fig. S18. Molecular mass estimation of VcaM4I and its variants. (A) Gel filtration of VcaM4I HNH domains in the absence of EVE domains. The molecular mass deduced from gel filtration experiments performed for VcaM4I HNH domains alone (carrying the inactivating H224A mutation enabling expression) and together with the linker helix confirms the dimer formation in the absence of EVE domains. **(B)** SDS-PAGE of wild-type VcaM4I and its variants confirms the molecular mass of the monomers.

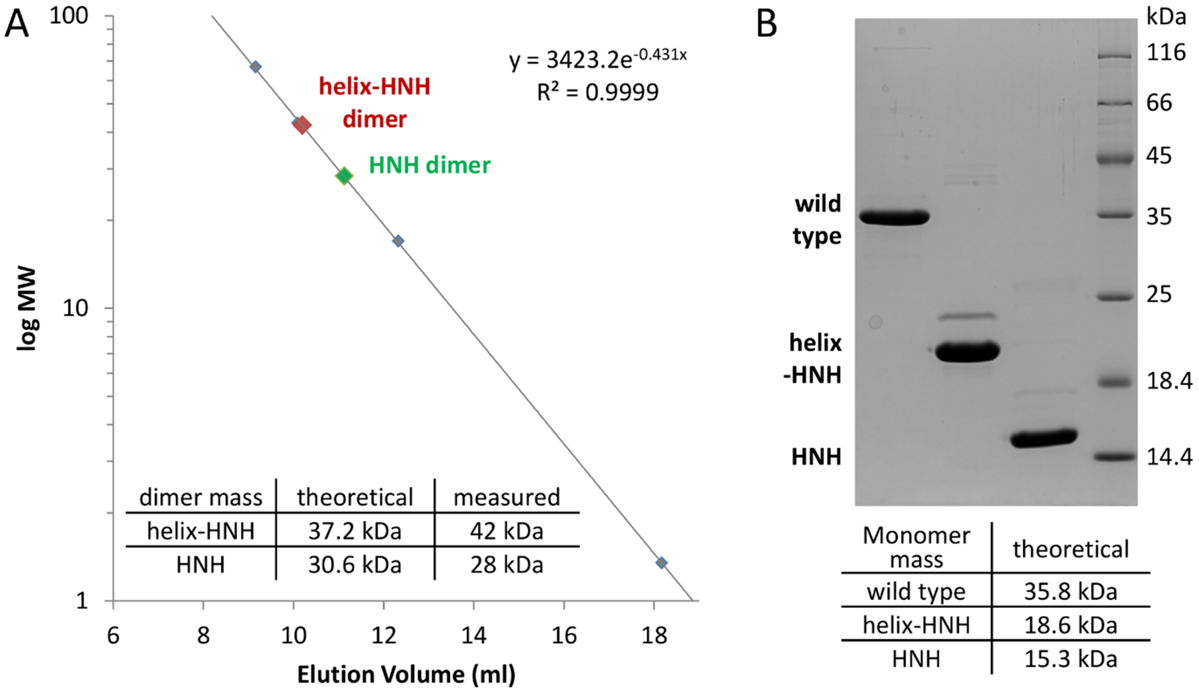

dimer mass	theoretical	measured
helix-HNH	37.2 kDa	42 kDa
HNH	30.6 kDa	28 kDa

Monomer mass	theoretical
wild type	35.8 kDa
helix-HNH	18.6 kDa
HNH	15.3 kDa

Suppl. Fig. S19. SAXS data for VcaM4I alone or in a stoichiometric mixture with 5mC 11-mer DNA. Experimental data (scatterplot) were recorded for VcaM4I with one dsDNA molecule for one EVE-domain per dimer. Theoretical scattering curves were calculated using CRYSOL program for Vcam4I alone (blue), VcaM4I with unbound dsDNA (cyan), VcaM4I with dsDNA bound to one EVE-domain (orange), a mixture of 50% VcaM4I alone and 50% VcaM4I with dsDNA bound to both EVE-domains (green), VcaM4I with dsDNA bound to the nuclease domain (based on a model, not crystallographic data, red), and VcaM4I with dsDNA bound to both EVE domains (black, as a negative control), as insufficient DNA was present for this scenario. The logarithm of the forward scattering was offset by 0, or an arbitrary value of -2, -4, -6, or -8, for clarity. The offset in the logarithm is equivalent to an overall scale factor for the data. The agreement between experimental and theoretical scattering curves is good for low protein or protein DNA concentrations as shown here (χ^2 values close to 1), but at higher concentration, there were systematic discrepancies between calculated and observed scattering curves (χ^2~7 for the highest 6.9 mg/ml tested concentration of VcaM4I with dsDNA).

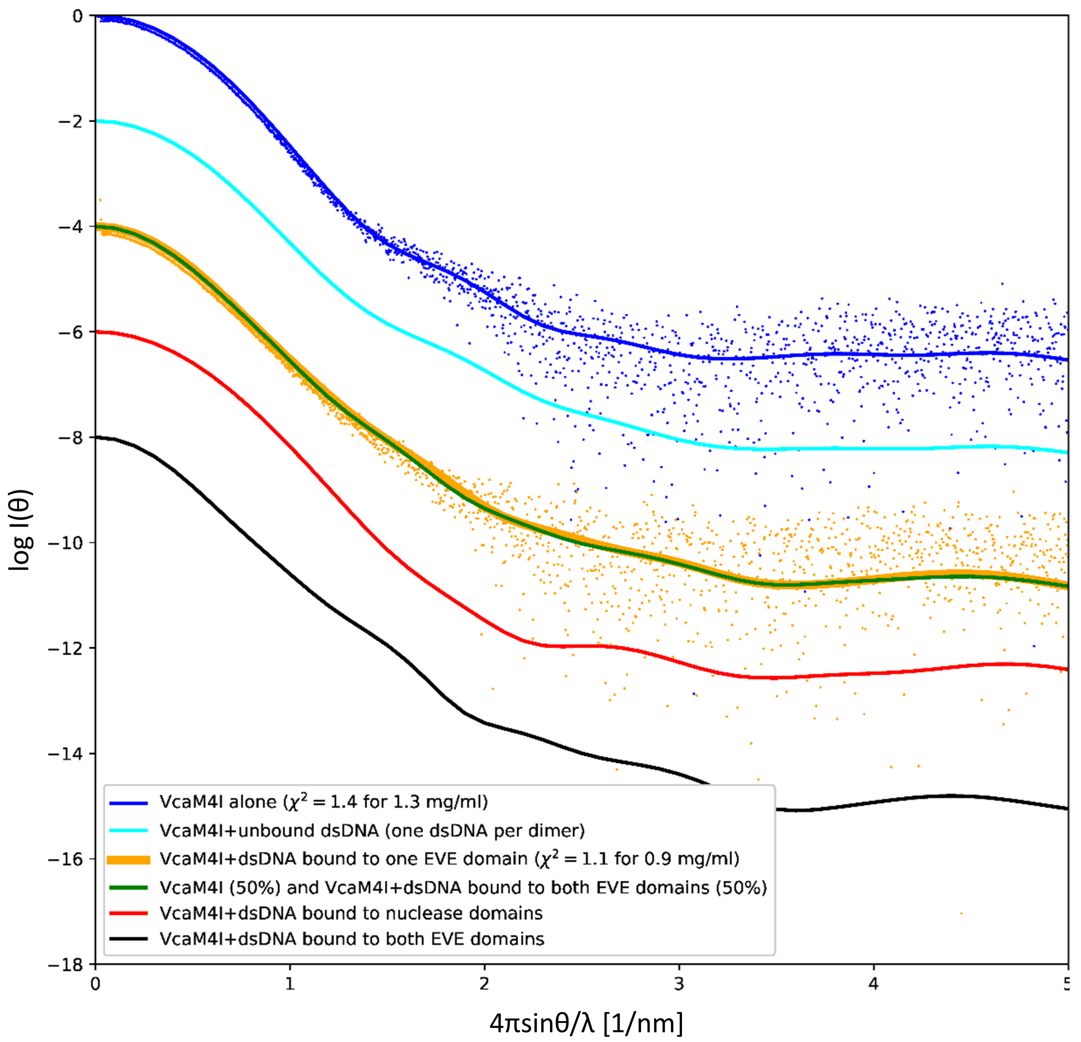

Suppl. Fig. S20. The model of the interaction of the THY28 EVE domain (PDB 5j3e, unpublished) with DNA. The DNA present in the crystal does not explore the putative 5hmC binding pocket, but after relatively minor adjustments the complex with the flipped-out base could be modelled. **(A)** The ribbon representation of the modelled complex in the orientation 90° rotated with respect to Fig. 2C. The protein is colored blue to red from N- to C- terminus. The C- terminal fragment outside of the canonical 5-stranded β-sheet is colored in light gray. **(B)** The residue conservation scores obtained with the help of the ConSurf (9) server and **(C)** electrostatic potential generated with the ABPS server (6) were mapped on the THY28 surface with CHIMERA (7). **(D,E)** Detailed view of the predicted THY28 flipped base binding pocket. The model does not unambiguously indicate the presence of the specificity determining residues. **(F)** The contacts with the potentially estranged guanine base.

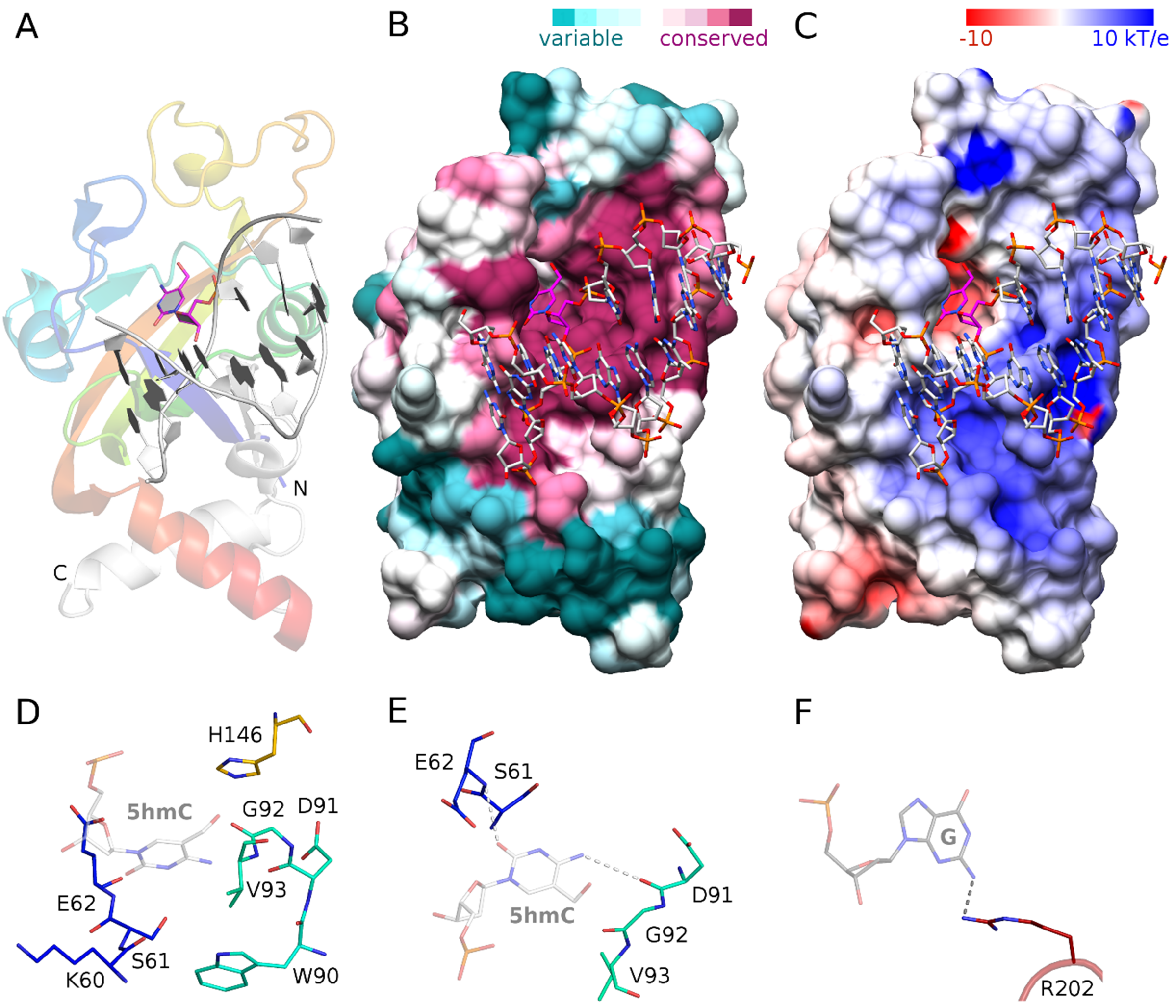

Supplementary Tables

Suppl. Table S1. Sequences of oligoduplexes used in this study. The red letters: “m” correspond to 5mC and “h” to 5hmC modified bases. 5hmC-containing oligos in duplexes 4, 5, and 6 for SAXS study were purchased from IDT.

5' TGACTGCAGGAAGGGGATCCG 3'<br>5' GTGATGGTGATGGTGGTGATGTTGAAGCTGCCACAAGGCAG 3'	Primers for **protein expression**
5' CCATG**m**GCTGA 3'<br>3' GGTACGCGACT 5'	11-mer dsDNA used for **crystallography & SAXS**<br>m=5mC
Cy5 CCATGCAGCTGACTACTGTCCTGTCGGTCACC 3'<br>3' GGTACGTCGACTGATGACAGGACAGC**x**AGTGG 5'	32-mer used for **EMSA,** x=5mc or 5hmC
5' CCATG**h**AGCTGACTACTGTCCTGTCGGTCACC 3'<br>3' GGTACGTCGACTGATGACAGGACAGC**h**AGTGG 5'	32-mer dsDNA used for **SAXS,** h=5hmC
5' CCATG**h**AGCTGATCTACTGTCCGAATGCTGATCCC 3'<br>3' GGTACGTCGACTAGATGACAGGCTTACGA**h**TAGGG 5'	35-mer dsDNA used for **SAXS,** h=5hmC
5' CCATG**h**AGCTGATGATCTACTGTCCGAGCAACTGGCAGC 3'<br>3' GGTACGTCGACTACTAGATGACAGGCTCGTTGA**h**CGTCG 5'	39-mer dsDNA used for **SAXS,** h=5hmC

Suppl. Table S2. Predicted structural homologs of the VcaM4I EVE domain. The DALI server (5) was used to pinpoint the proteins that are most structurally similar to the VcaM4I EVE domain. The subset of the structures deposited in the PDB used for comparison excludes the entries with proteins that share more than 90% of sequence identity. The structures with similarity defined by DALI Z-score > 5 are listed in the table. Only selected GO terms are shown.

PDB ID	DALI Z-score	name	Description		Molecular function			Inverse ec2go, kegg2go		
			Estimated PPV, description		Estimated PPV, GO-i d, description					
2HD9	14.3	UPF0310 PROTEIN PH1033	0.61	EVE domain-containing protein						
2P5D	13.9	UPF0310 PROTEIN MJECL36	0.72	EVE domain-containing protein						
2EVE	12.4	HYPOTHETICAL PROTEIN PSPTO5229	0.76	EVE domain-containing protein						
2G2X	12.4	HYPOTHETICAL PROTEIN PP5205	0.66	EVE domain						
2AR1	12.4	HYPOTHETICAL PROTEIN	0.51	EVE domain containing protein	0.47	GO:0003677	DNA binding	0.42	EC:1.14	GO:0016705
					0.42	GO:0016705	oxidoreductase activity, acting on paired donors, with incorporation or reduction of molecular oxygen			
					0.4	GO:0020037	heme binding			
1ZCE	12.2	HYPOTHETICAL PROTEIN ATU2648	0.61	EVE domain-containing protein						
5J3E	11.8	THYMOCYTE NUCLEAR PROTEIN 1 (THY28)	0.94	Thymocyte nuclear protein 1						
2GBS	11.7	HYPOTHETICAL PROTEIN RPA0253	0.55	Ubiquinol-cytochrome c reductase						
4WQN	10.5	YTH DOMAIN CONTAINING FAMILY PROTEIN 2	0.73	YTH domain containing family protein 2	0.63	GO:1990247	N6-methyladenosine-containing RNA binding			
2YU6	10.2	YTH DOMAIN CONTAINING PROTEIN 2	0.52	YTH domain containing family protein 2	0.71	GO:0003724	RNA helicase activity	0.71	EC:3.6.4.13	GO:0003724
					0.69	GO:1990247	N6-methyladenosine-containing RNA binding			
					0.61	GO:0008186	RNA-dependent ATPase activity			
					0.6	GO:0070063	RNA polymerase binding			
5ZUU	10.2	YTH DOMAIN CONTAINING FAMILY PROTEIN 2	0.53	Cleavage and polyadenylation specificity factor CPSF30	0.61	GO:1990247	N6-methyladenosine-containing RNA binding	0.35	EC:3.1	GO:0016788
					0.56	GO:0003677	DNA binding			
					0.52	GO:0046872	metal ion binding			
					0.47	GO:0003729	mRNA binding			
4RCM	10	METHYLATED RNA BINDING PROTEIN 1	0.38	YTH-domain-containing protein	0.53	GO:1990247	N6-methyladenosine-containing RNA binding			
					0.5	GO:0003730	mRNA 3'-UTR binding			

4U8T	10	ZYRO0G01672P	0.37	YTH-domain-containing protein	0.43	GO:1990247	N6-methyladenosine-containing RNA binding			
					0.4	GO:0003730	mRNA 3'-UTR binding			
5GUQ	9.8	HELIX TURN HELIX DOMAIN CONTAINING PROTEIN	0.4	ASCH domain-containing protein						
6N0S	9.8	YTH DOMAIN CONTAINING PROTEIN 1	0	Uncharacterized protein						
4RCJ	9.5	YTH DOMAIN CONTAINING FAMILY PROTEIN 1	0.58	YTH DOMAINCONTAINING FAMILY PROTEIN 1	0.6	GO:1990247	N6-methyladenosine-containing RNA binding			
					0.5	GO:0043022	ribosome binding			
6FPXA	9.1	YTH DOMAIN CONTAINING PROTEIN MMI1	0	Uncharacterized protein	0.88	GO:1905762	CCR4-NOT complex binding			
					0.86	GO:0097157	pre-mRNA intronic binding			
					0.63	GO:1990247	N6-methyladenosine-containing RNA binding			
					0.63	GO:0003729	mRNA binding			
2YUD	8.8	YTH DOMAIN CONTAINING PROTEIN 1	0	Uncharacterized protein						
6P0F	6.6	GTPASE SUBUNIT OF RESTRICTION ENDONUCLEASE	0.23	GTPase subunit of restriction endonuclease	0.65	GO:0004519	endonuclease activity	0.61	EC:3.6.1.3	GO:0016887
					0.61	GO:0016887	ATPase activity			
2KKU	6.4	UNCHARACTERIZED PROTEIN	0.95	DUF365 domain-containing protein						
3IUW	6.3	ACTIVATING SIGNAL CO-INTEGRATOR	0.57	ASCH domain-containing protein						
2Z0T	6.2	PUTATIVE UNCHARACTERIZED PROTEIN PH0355	0.79	ProFAR isomerase associated superfamily protein	0.63	GO:0016853	isomerase activity	0.63	EC:5	GO:0016853
6KIR	6.1	UNCHARACTERIZED PROTEIN CXORF40 HOMOLOG	0.59	ASCH domain-containing protein						
2DP9	5.8	HYPOTHETICAL PROTEIN TTHA0113	0.66	Asch domain superfamily						
1XNE	5.6	HYPOTHETICAL PROTEIN PF0469	0.62	ProFAR isomerase associated superfamily protein	0.63	GO:0016853	isomerase activity	0.63	EC:5	GO:0016853
2E5O	5.5	ACTIVATING SIGNAL COINTEGRATOR 1	0.46	Activating signal cointegrator 1	0.63	GO:0008270	zinc ion binding			
					0.58	GO:0035035	histone acetyltransferase binding			
					0.57	GO:0030331	estrogen receptor binding			
					0.55	GO:0061629	RNA polymerase II-specific DNA-binding transcription factor binding			
5Y7D	5.3	PROTEIN CXORF40A	0.59	ASCH domain-containing protein						
1S04	5.3	HYPOTHETICAL PROTEIN PF0455	0.79	ProFAR isomerase associated superfamily protein	0.63	GO:0016853	isomerase activity	0.63	EC:5	GO:0016853
2GKS	5.2	BIFUNCTIONAL SAT/APS KINASE (CYSC)	0.59	Bifunctional sulfate adenylyltransferase/adenylylsulfate kinase	0.74	GO:0004020	adenylylsulfate kinase activity	0.74	EC:2.7.1.25	GO:0004020
					0.71	GO:0004781	sulfate adenylyltransferase (ATP) activity			

Suppl. Table S3. Summary of DNA cleavage activity and *in vivo* phage restriction activity.

VcaM4I variant	Cleavage activity	Restriction of T4gt (no IPTG)	Restriction of T4gt (+ IPTG)	Note
WT	+	+	+++	
T11A	+ (?)	-	-	Conflicting activity *in vivo* vs *in vitro*. Inconclusive.
E15A	+/-	+	++	Attenuated activity
W22A	not tested	- (?)	- (?)	Inconclusive (due to a frame shift mutation in addition to W22A)
V77A	+	+	+++	
G79A	+	+	++	
D81A	+	+	++	
W82A	+	+	++	
D81A/W82A	+	+	++	protein made at 16 °C
Q128A	+	+	++	
Y130A	+/-	-	- (poor cell lawn)	lower protein yield, toxic to cells
H224A	-	-	+	catalytic residue
N241A	-	-	+	no protein made at 18 °C or 16°C
D250A	-	-	+	
H251A	-	-	+	
D254A	-	-	+	

+, active; -, inactive; +/-, attenuated activity, ?, inconclusive.

Supplementary Raw Data

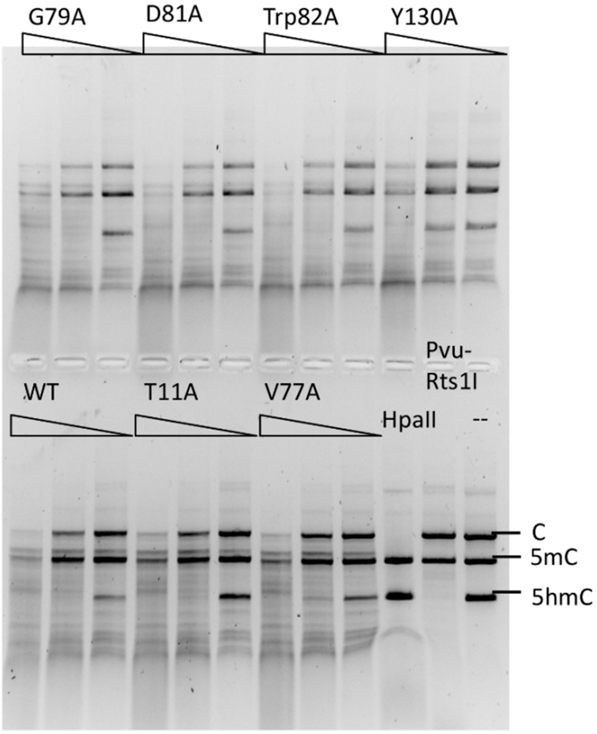

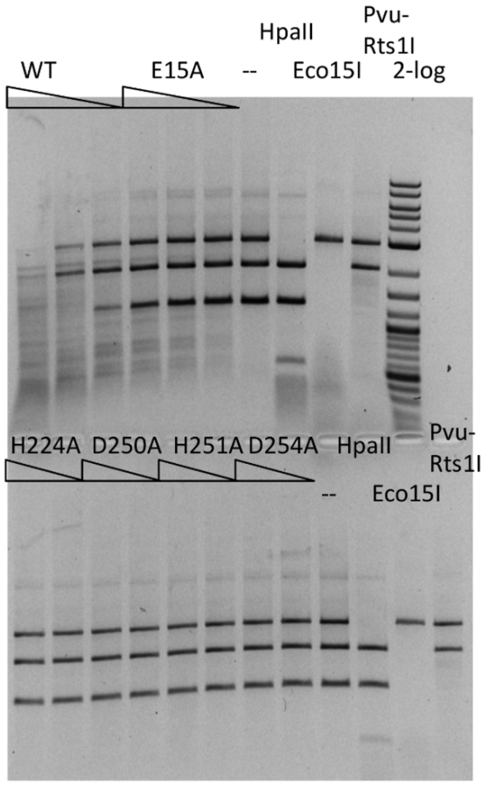

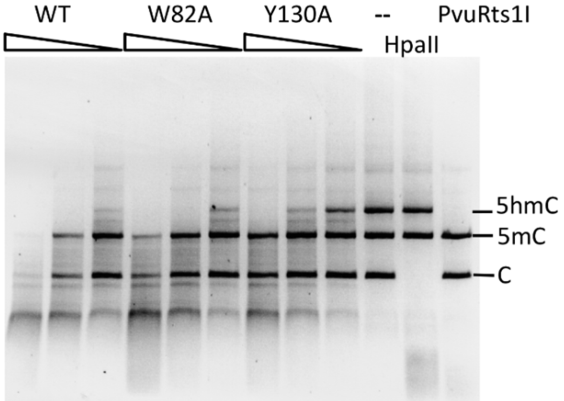

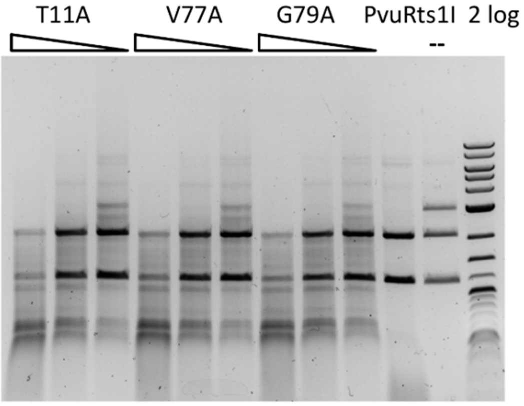

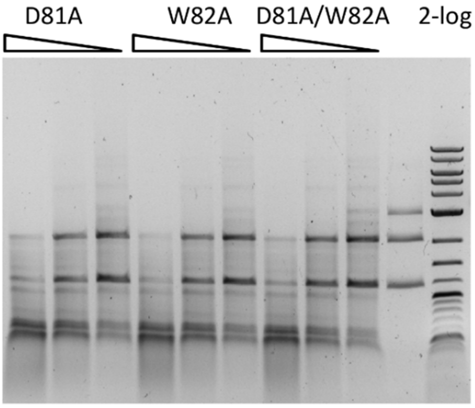

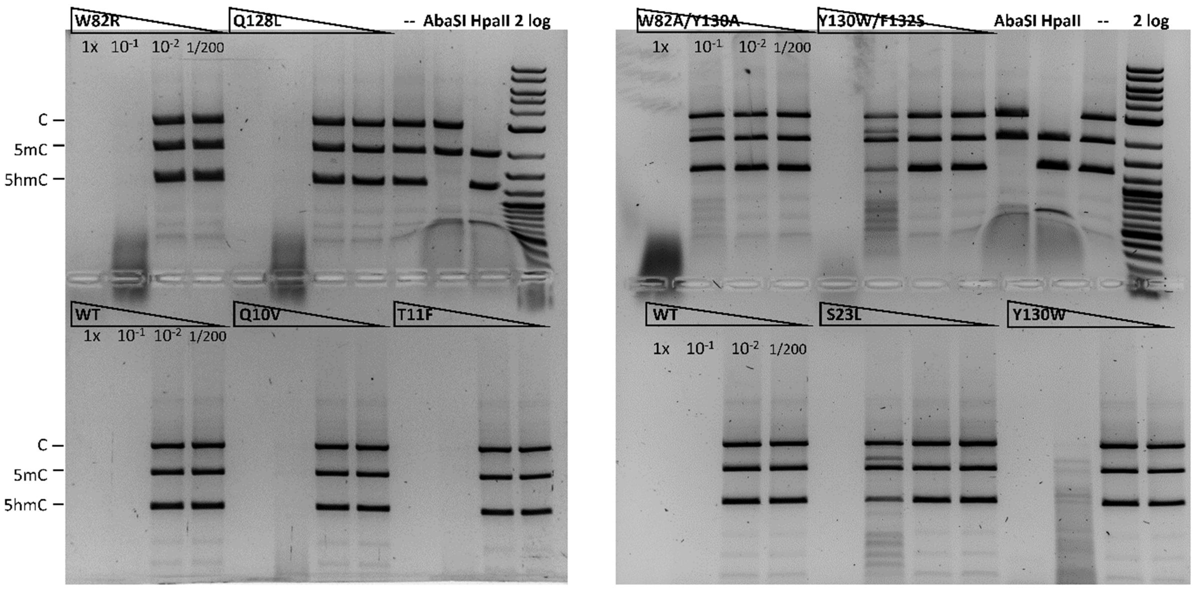
W82R
Q128L
-- AbaSI HpaII 2 log
1x 10-1 10-2 1/200
C
5mC
5hmC
WT
Q10V
T11F
1x 10-1 10-2 1/200
C
5mC
5hmC
W82A/Y130A
Y130W/F132S
AbaSI HpaII -- 2 log
1x 10-1 10-2 1/200
WT
S23L
Y130W
1x 10-1 10-2 1/200

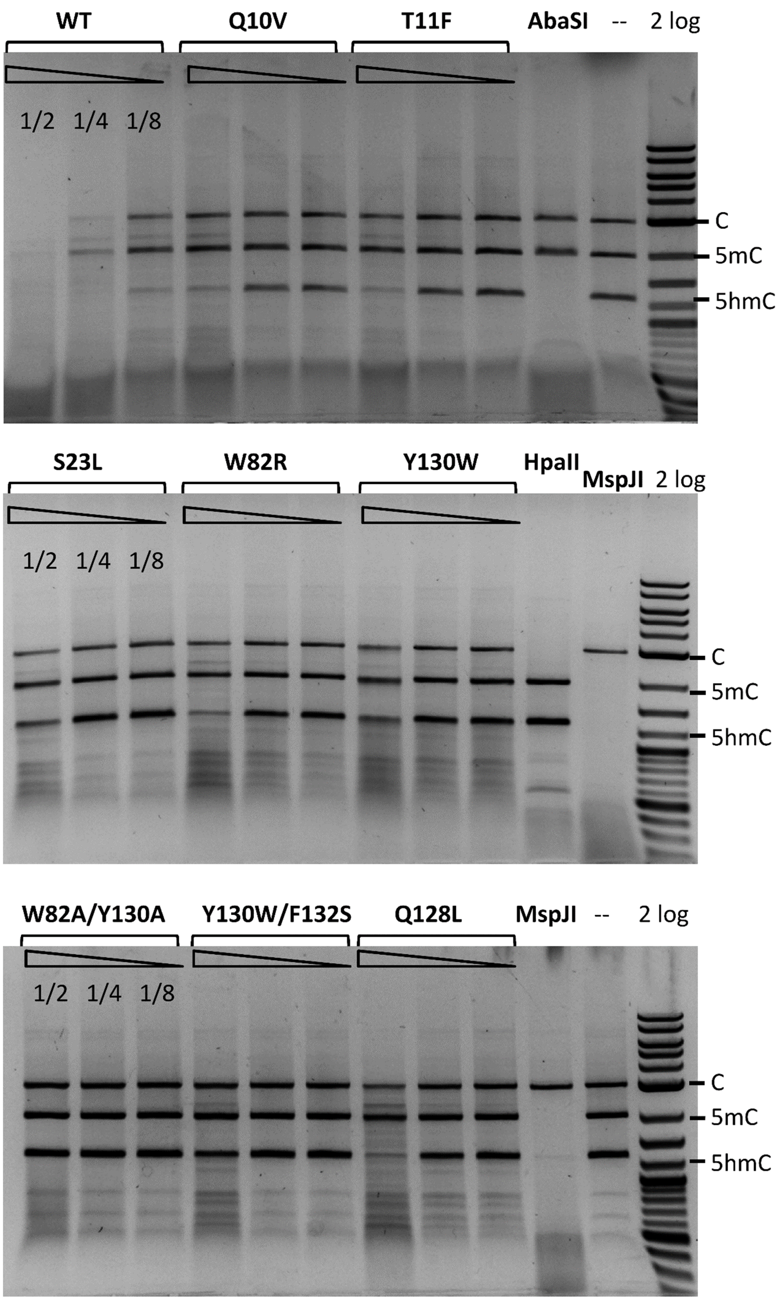
WT
Q10V
T11F
AbaSI
--
2 log
1/2 1/4 1/8
C
5mC
5hmC
S23L
W82R
Y130W
HpaII
MspJI
2 log
1/2 1/4 1/8
C
5mC
5hmC
W82A/Y130A
Y130W/F132S
Q128L
MspJI
--
2 log
1/2 1/4 1/8
C
5mC
5hmC

References

1. Kisiala, M., Copelas, A., Czapinska, H., Xu, S.Y. and Bochtler, M. (2018) Crystal structure of the modification-dependent SRA-HNH endonuclease TagI. *Nucleic Acids Res*, **46**, 10489-10503.
2. Czapinska, H., Kowalska, M., Zagorskaite, E., Manakova, E., Slyvka, A., Xu, S.Y., Siksnys, V., Sasnauskas, G. and Bochtler, M. (2018) Activity and structure of EcoKMcrA. *Nucleic Acids Res*, **46**, 9829-9841.
3. Liu, G., Fu, W., Zhang, Z., He, Y., Yu, H., Wang, Y., Wang, X., Zhao, Y.L., Deng, Z., Wu, G. *et al.* (2018) Structural basis for the recognition of sulfur in phosphorothioated DNA. *Nat Commun*, **9**, 4689.
4. Sokolowska, M., Czapinska, H. and Bochtler, M. (2009) Crystal structure of the beta beta alpha-Me type II restriction endonuclease Hpy99I with target DNA. *Nucleic Acids Res*, **37**, 3799-3810.
5. Holm, L. and Laakso, L.M. (2016) Dali server update. *Nucleic Acids Res*, **44**, W351-355.
6. Jurrus, E., Engel, D., Star, K., Monson, K., Brandi, J., Felberg, L.E., Brookes, D.H., Wilson, L., Chen, J., Liles, K. *et al.* (2018) Improvements to the APBS biomolecular solvation software suite. *Protein Sci*, **27**, 112-128.
7. Pettersen, E.F., Goddard, T.D., Huang, C.C., Couch, G.S., Greenblatt, D.M., Meng, E.C. and Ferrin, T.E. (2004) UCSF Chimera--a visualization system for exploratory research and analysis. *J Comput Chem*, **25**, 1605-1612.
8. Lutz, T., Flodman, K., Copelas, A., Czapinska, H., Mabuchi, M., Fomenkov, A., He, X., Bochtler, M. and Xu, S.Y. (2019) A protein architecture guided screen for modification dependent restriction endonucleases. *Nucleic Acids Res*, **47**, 9761-9776.
9. Ashkenazy, H., Abadi, S., Martz, E., Chay, O., Mayrose, I., Pupko, T. and Ben-Tal, N. (2016) ConSurf 2016: an improved methodology to estimate and visualize evolutionary conservation in macromolecules. *Nucleic Acids Res*, **44**, W344-350.
